# Alternative and Sustainable Protein Sources in Pig Diet: A Review

**DOI:** 10.3390/ani14020310

**Published:** 2024-01-19

**Authors:** Antonia Lestingi

**Affiliations:** Department of Veterinary Medicine, University of Bari Aldo Moro, Valenzano, 70010 Bari, Italy; antonia.lestingi@uniba.it

**Keywords:** pigs, insect larvae, spirulina, rapeseed meal, grain legumes, nutritional value

## Abstract

**Simple Summary:**

Nutritional and anti-nutritional factors of Spirulina, insect larvae such as *Tenebrio molitor* and *Hermetia illucens*, rapeseed meal, and grain legumes such as fava beans, peas, lupins, and chickpea are reviewed, in order to re-evaluate the use of these potential protein ingredients in pig diets. The effects on pig performance, digestion, immune system, and the physicochemical and sensorial characteristics of pork are updated. The limits of their use to be accounted for in pig diet formulation are revisited, together with the possible treatments to improve their nutritional value.

**Abstract:**

The search for alternative protein sources to soybean meal (SBM) in animal feeding is a strategic objective to reduce production costs and contribute to sustainable animal production. Spirulina, due to the high protein content, has emerged as a potential cost-effective, sustainable, viable, and high-nutritional-value food resource for many animal species. Insect larvae (*Tenebrio molitor* and *Hermetia illucens*) are also considered potential alternatives to SBM, given their high edible percentage of almost 100%, as well as a protein value higher than that of vegetable proteins. Rapeseed meal and grain legumes, such as fava beans, peas, lupins, and chickpea, can also be used as locally producible protein ingredients. This study reviews the nutritional value of these potential alternatives to SBM in pig diets, and their effects on animal performance, digestion, immune system, and the physicochemical and sensorial characteristics of meat, including processed pork products. The limits on their use in pig feeding are also reviewed to indicate gaps to be filled in future research on the supplementation level of these potential alternative protein sources in pig diets.

## 1. Introduction

Pork consumption has been predicted to increase by 105% between 2010 and 2050 [1], due to the increase in the world population [2,3]. The main protein ingredients in diets for monogastrics (chickens and pigs) are currently fish meal, processed animal proteins, milk by-products, soybean meal (SBM), rapeseed meal, and canola meal [3,4,5]. SBM is used in compound feed for pig farming at a maximum inclusion level of 18%. In a previous review, the sustainability problems of pig farming and the need to mitigate the environmental impact of pork production were highlighted [6]. In recent years, genetically modified soy imported into Europe has increased and its price has fluctuated significantly [2,3]. Therefore, the scientific community is currently intensifying its efforts in finding alternative protein sources to soya bean in animal diets in order to meet the demand for high-quality and sustainable proteins [7]. A new food resource should ideally be characterized by a high nutritional value and feed conversion efficiency, but also be able to provide high-quality animal products, using land and water efficiently [8]. The high economic and environmental costs associated with the production and long-distance transportation of feedstuffs such as cereals and soybeans, mainly produced in North and South America, and their direct competition with human consumption, have important repercussions on the sustainability of food and livestock production [9,10]. Furthermore, the search for alternative, long-term, and low-cost protein and energy sources for use in farm animal diets has intensified, becoming a strategic objective after the COVID-19 pandemic [11].

Edible insects (entomophagy) are receiving attention as an alternative sustainable nutritional source for both humans and animals [2], thanks to the advantage that they may be produced without the use of arable land and are rich in proteins [12]. Indeed, insect larvae use less water and land, and fewer resources for their production compared to conventional plant-based ingredients used for feed [12,13]. In general, insects have an advantageous food conversion ratio and are be able to convert low-value organic waste into high-value protein, that could replace soybean meal and fishmeal, available in increasingly limited quantities [14]. According to Hong et al. (2020) [2], the use of edible insects in animal feed is expected to increase in the future. As a result, many companies, such as in France and China, have undertaken the large-scale production of insects, both for food and feed, around the world. Based on these predictions, the price and use of insects in diets will be achievable and expand, respectively. Insects have several advantages as the percentage of edible part is almost 100% [2,15], and their nutritional value is much higher than that of plants in terms of proteins, essential amino acids, vitamins, and minerals [2]. Makkar et al. (2014) [14] reported that insects may have an essential amino acid composition that matches the requirements of growing pigs and broiler chickens. Indeed, insects, in general, contain high quantities of lysine, threonine, and methionine, the main limiting essential amino acids (AAs) in low-protein diets based on cereals and legumes for pigs and poultry. It has been reported that insects, as well as being an alternative protein source, are also rich in fat, especially full-fat black soldier fly larvae (*Hermetia illucens* L.) (BSFL), and can also be a source of net energy (NE) in pig diets [12,16].

In recent years, the use of blue-green algae has also become widespread as biologically active food supplements in both human and animal nutrition [17,18,19,20]. They are used in human and animal nutrition, in cosmetics, and in the production of valuable substances (e.g., fatty acids and pigments) [21]. It has been reported that microalgae, due to the high protein, carbohydrate, and fat content, are comparable or even superior to conventional feedstuffs, such as soybean meal [22]. Spirulina (*Arthrospira* sp.) has emerged as a potential cost-effective, sustainable, viable, and high-nutritional-value food resource for many animal species [7]. It has been reported that the direct intake of Spirulina can improve growth performance and meat quality in ruminants, chickens, pigs, and rabbits [7,11,23].

Rapeseed meal, legume grains (including chickpea, pea, lupin, and fava bean), and other plant seeds can be a source of both energy and protein in animal diets [24,25,26,27]. Rapeseed meal (RSM) can represent an alternative to SBM in monogastric diets as it is an abundant and economical by-product of oil and biofuel production [28,29,30]. China, India, Canada, Europe, and Australia are the regions where rapeseed is mainly grown [28]. Legume grains are under cultivation in a considerable area worldwide and are an excellent low-fat source of protein, dietary fiber, starch, micronutrients, and phytochemicals [25,26,27,31,32]. Rapeseed meal (RSM) and legume seeds have been proposed as possible alternative protein sources as high-protein ingredients that can be produced in Europe, contributing to more sustainable pork production [5,33,34]. Germany, France, and Finland are adopting a common strategy to encourage the extensive cultivation and production of legumes as excellent potential protein sources, to reduce the cost of diets while maintaining production efficiency [35].

This review focuses on the nutritional values of these potential protein sources (micro algae, insect larvae, rapeseed meal, and grain legumes) and on their effects on growth performance, carcass yield, meat quality, digestion, and immunity when used in pig diets.

## 2. Microalgae

### 2.1. Nutritional Value

Among microalgae, spirulina (*Arthrospira platensis*) is the most used in the production of food, both for humans and livestock [9]. *Spirulina platensis* and the freshwater microalgae *Chlorella vulgaris* are often indicated in human nutrition for their beneficial actions on intestinal health, such as antioxidant, anti-inflammatory, and antimicrobial activities [36,37].

Spirulina is an edible blue-green filamentous, spiral-shaped microalga of the cyanobacteria phylum and is therefore an autotrophic prokaryote [9,38]. It is naturally present in the alkaline lakes of Mexico and Africa, where it has historically been a source of food for the ancient inhabitants of those places [7].

Several authors have been dealing with the nutritional properties of spirulina (Table 1) for some time [39,40,41,42].

Spirulina has a high nutritional value, in particular a high protein content (from 50% to 70% of dry weight) and an interesting lipid content (from 5% to 14% of dry weight) [37,43,44]. It has a unique composition due to its balanced content of amino acids, vitamins, minerals, pigments, carotenoids, chlorophyll, and essential polyunsaturated fatty acids, such as γ-linolenic acid (GLA), which has beneficial health effects [7,23,38]. It also contains high quantities of phycocyanin (27%) [17].

Commercial production of spirulina may occur using a nutrient-rich liquid medium with high efficiency in land use [7]. Spirulina has been reported to outperform many other traditional types of animal feed, such as wheat, corn, barley, and soy, in terms of protein production per unit of land [45,46]. Furthermore, desalinated waste water [47] and animal feces can be actively used for its production in order to improve the replication medium. This has been reported with feces from pigs [48] and cattle [49], with results demonstrating the safety of administering Spirulina to livestock [50].

However, microalgae cell wall carbohydrates are very difficult to digest and utilize by monogastric animals, such as pigs [9]. The cell wall of microalgae has a complex structure and composition that has not been explored in depth, although the presence of a polymeric matrix has been reported, made up of cellulose and a further trilaminar sheath in which there is a substance resistant to enzymatic degradation, the algaenan [51,52].

It has been hypothesized that Spirulina, integrated with active carbohydrate enzymes (CAZymes) capable of degrading the complex polysaccharides of the cell wall, can be usefully used to facilitate the access of digestive enzymes to the cell content, improving nutrient digestibility [9]. Among the enzymes used is lysozyme, a CAZyme capable of degrading the peptidoglycan constituent of the cell walls of prokaryotes, thus making proteins and pigments accessible to the enzymes of the digestive system [9,53].

### 2.2. The Use of Spirulina in Pig Diets

Several studies focus on the use of Spirulina in piglet diets as a supplement [17,19,54]. The first results on the use of spirulina in pig diets are contrasting [7]. As already argued by Holman and Malau-Aduli (2013) [7], the different results obtained in the growth of pigs fed diets supplemented or not with spirulina are attributable to the different experimental procedures, i.e., to the genetics and state of health of the animals, the composition of the diet, and the level and form of inclusion of spirulina in the diet, pelleted or not [19].

In pigs, Spirulina and Chlorella have been studied as an alternative to antibiotic use to ensure gut health after weaning, limiting intestinal damage caused by immune and inflammatory responses to dietary transition and oxidative stress [55,56]. Studies highlighted a potential effect of Spirulina and Chlorella supplementation on intestinal development, and the prevention of mild digestive disorders with a reduction of sick animals [17,54]. However, results are not conclusive and the authors see the need for further investigations to determine the mechanism of action of Spirulina and Chlorella on the health and physiology of the intestine.

Furthermore, research has addressed the enrichment of microalgae biomass with microelements through biosorption and bioaccumulation, in order to produce dietary supplements for animals, as is done for humans [57]. In the study by Saeid et al. (2013) [57], the effect of the use of the microalgae *Spirulina maxima*, enriched with copper (Sm-Cu, as a replacement for inorganic salts), on performance and the metabolic and physiological values of fattening pigs is observed. The study reports a lowering of LDL cholesterol (by 23%) and total cholesterol (by 10.5%) in blood serum, as well as an increase in the parameter a* in the meat from pigs fed Sm-Cu diets.

Simkus et al. (2013) [58] observed the growth performance and meat quality of Landrace and Yorkshire fattening pig crossbreds fed daily and individually with 2 g of fresh blue algae biomass *Spirulina platensis* at 75% moisture mixed with forage. The average daily weight gain of pigs fed fresh Spirulina biomass was 9.26% higher, with 100 kg of weight being reached 7.37 days faster, and the food energy consumed per 1 kg increase of weight was 1.28 MJ lower than that of pigs in the control group. The carcass weight of the experimental pigs was 2.02% higher and the amount of intramuscular fat 0.33% lower compared to the control group. Color, pH, cooking loss, and tenderness were not affected by dietary treatments.

The effects of Spirulina supplementation on the performance, carcass, and meat quality of pigs, when administered not directly but to their gestating and lactating mothers until weaning, have recently been studied [38]. In particular, Lugarà et al. (2022) [38] studied the long-term influence of maternal spirulina dietary supplementation (no supplementation and 20 g of spirulina per day in tablets) and the energy density of the diet (control and high-energy density, HED), throughout gestation and lactation until weaning, on growth performance, carcass characteristics, meat quality, and fatty acid profile, in both male and female pigs. The daily weight gain during the entire fattening period (4 months) was reduced (−7.4%) in males from mothers who had received spirulina supplementation compared to males from sows who had not received supplementation, even if the slaughter weight did not differ between the two groups. The daily gain, slaughter weight, and fattening period were not influenced by maternal spirulina supplementation in females. No significant differences were found regarding feed conversion rate, feed intake, and carcass characteristics in pigs of either sex from mothers with and without dietary spirulina supplementation. The dietary treatment did not influence physico-chemical meat characteristics. Maternal spirulina supplementation tended to improve the polyunsaturated fatty acid (PUFA)/saturated FA (SFA) ratio in the intramuscular fat, without any influence on the n-6/n-3 FA ratio. The results of this study highlight a sex-specific response of offspring growth following maternal spirulina supplementation.

Nedeva et al. (2014) [17] evaluated the supplementation of 0.15% and 0.2% spirulina in the diets of piglets (from 12.2–12.5 to 30.9–33.9 kg live weight) and observed an increase in growth performance together with a decrease in the feed conversion ratio. In contrast, Grinstead et al. (2000) [19] found minimal improvement in growth performance in piglets weaned on 0.2%, 0.5%, and 2% Spirulina for 28 days.

However, these different studies concern the use of microalgae in pig diets as supplements and not as feed ingredients.

The incorporation of microalga Spirulina as a feedstuff together with the supplementation of two exogenous enzymes was considered for the first time by Martins et al. (2021) [9]. The authors evaluated the effect of the use of Spirulina (10%) in post-weaning piglet diets (slaughtered at weights lower than the standard weight of 100 kg in order to obtain spit-roasted pigs), supplemented or not with two exogenous carbohydrate-degrading enzymes (CAZymes), a commercial mixture of CAZymes and lysozyme, on growth performance, digestibility, and meat quality in piglets. The use of 10% Spirulina in the piglets’ diet negatively affected growth performance, with a 9.1% decrease in final weight, a 14.2% decrease in average daily weight gain (ADG), and an 11.0% increase in the feed conversion ratio (FCR), compared to the control group. An increase in the viscosity of the digesta and a lower total tract apparent digestibility (TTAD) of crude protein were observed as a result of their resistance to piglets’ endogenous proteolytic enzymes. Indeed, the authors of the study, in agreement with what was reported by Evans et al. (2015) in chickens [59], argue that the strong increase in the viscosity of the digesta of piglets that had consumed the microalgae was likely the result of the gelation of poorly digestible proteins of spirulina. Furthermore, lysozyme supplementation increased the TTAD of crude fat and acidic detergent fiber, compared to the *Spirulina* supplemented group and the control group, respectively. In the experimental conditions of these Authors, the use of exogenous enzymes in piglet diets did not improve digestibility of *Spirulina* proteins nor does it prevent their gelation. Meat quality was not negatively affected by the addition of *Spirulina* in the piglets’ diet, either alone or associated with enzymes. Contrary to what has been reported by other authors on the antioxidant activity of *Spirulina* [37,43], no protective effect against meat lipid oxidation was observed during the 7-day storage period.

Interestingly, an improvement in sperm quality (11% increase in volume, and 5% increase in motility and vitality after conservation) has been reported in wild boars that had received a dietary Spirulina extract compared to those fed an un-supplemented diet [60]. This would be of interest for the revaluation of this species breeding [6].

The effects of Spirulina supplementation in pig diets are presented in Table 2.

## 3. Insect Larvae

### 3.1. Nutritional Value of Insect Larvae

Yellow mealworm (*Tenebrio molitor*), black soldier fly (BSF) (*Hermetia illucens*), and common housefly (*Musca domestica*) are the insect species that could potentially be produced on a large scale for use as protein ingredients in animal diets [2,3].

**Figure 1:**
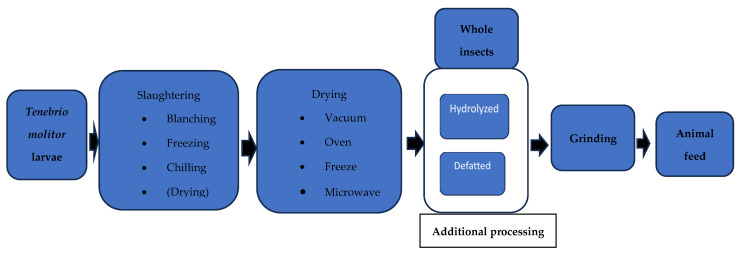
Schematic representation of processes that *Tenebrio molitor* larvae undergo before animal consumption [2]

*Tenebrio molitor* larvae, also known as mealworm or yellow mealworm, have good nutritional value, thanks to their protein and fat contents [61,62,63], their digestibility [64,65], flavor [66], and functionality, given by chitin and antimicrobial peptides (AMPs) [2,67]. Hong et al. (2020) [2] recently reviewed studies regarding *Tenebrio molitor* larvae as an alternative protein source in monogastric diets. The crude protein content (dry matter basis) of *T. molitor* larvae averages 52.4% [2] ranging from 47.0 [67] to 60.2% [68], being higher than that of conventional SBM (44.0%) [69], although lower than that of fishmeal (67.5%) [69]. The amino acid profile is of high quality, to be considered a highly sustainable protein source alternative to SBM or fishmeal [2]. Fiber is found in their cuticles and varies from 4.19% [70] to 22.35% [68]. The crude fat content (dry matter basis) averages 30.8% [2], ranging from 19.1% [68] to 37.7% [62], and can vary depending on whether it has been defatted or not (Figure 1) [2]. Regarding the fatty acid composition of *Tenebrio molitor* larvae (DM basis), it has been reported that the percentages of SFA and unsaturated fatty acids range from 22.2% [71] to 23.3% [68] and from 77.7% [71] to 79.0% [72], respectively. Essential polyunsaturated fatty acids (PUFA), from the n-3 and n-6 series, are also detected [2]. However, *T. molitor* larvae can undergo not only a defatting treatment but also hydrolysis before grinding (Figure 1) [2].

*T. molitor* larvae contain various minerals such as calcium, phosphorus, sodium, potassium, magnesium, iron, zinc, and copper. Among microelements, the most represented are iron (63.0–100.0 mg/kg) and zinc (102.0–117.4 mg/kg) [68,71]. It is reported that *Tenebrio molitor* larvae are easy to raise and feed, as they have a stable protein content, independent of their diet [2]. Thanks to these characteristics, they have been industrially produced as feed for pets, zoo animals, and livestock [2].

Lu et al. (2022) [3] recently reviewed studies regarding the composition of black soldier fly larvae (*Hermetia illucens* L., BSFL) and their potential use as an alternative protein source in animal diets. BSFL are also reported as a good source of proteins (216–655 g/kg of crude protein on a dry matter basis for defatted BSFL) and essential amino acids, being also rich in other nutrients such as fats (298–515.3 g/kg of crude fat on a dry matter basis) and minerals (27–132 g/kg of ash on a dry matter basis for full-fat BSFL) [3]. Moreover, BSFL are a potentially source of antimicrobial peptides (AMPs) that are produced as a reaction against invading pathogens, with a broad-spectrum action on both Gram-positive and Gram-negative bacteria [73]. It is known that BSFL have a high content of SFA (362–782.9 g/kg on a dry matter basis) while that of PUFA is usually low [74]. BSFL have a high concentration of the medium-chain fatty acid lauric acid C12:0 (75–575.6 g/kg on a dry matter basis), a natural antimicrobial that acts in particular against Gram-positive bacteria [74]. However, the substrate on which larvae are reared significantly influences their fatty acid composition, i.e., the overall synthesis of fatty acids from the n-3 and n-6 series [74]. The calcium to phosphorus ratio varies depending on whether the BSFL meal is defatted or not, and this requires attention in the formulation of pig diets, in order to ensure appropriate calcium to phosphorus ratios in the complete feed and avoid antagonisms among minerals [75]. Rigorous quality controls of BSFLM are required from suppliers [12].

BSFL can be easily grown and spread on any nutrient substrate, which can be represented by vegetation residues, manure, animal waste, food scraps, agricultural by-products, or straw [12,16,76], and their composition is influenced by the growth substrate [16]. The ability of BSFL to grow on organic waste substrates makes them more sustainable protein sources for preparing pig diets. It is reported that 1 kg of BSFL biomass can be obtained per 2 kg of growth substrate [14], thus reducing the organic matter discharged to landfills [12]. In Spranghers et al.’s study (2016) [16], BSFL were grown on four different substrates such as chicken feed, vegetable waste, biogas digestate, and restaurant waste, with the aim of evaluating their influence on the amino acid, fatty acid, and mineral composition. Prepupae protein content ranged between 399 and 431 g/kg (on a dry matter basis) among experimental groups, with minimal differences found in the amino acid composition, due to the growth substrate. Conversely, prepupae reared on biogas digestate showed lower ether extract (EE) and higher ash (218 and 197 g/kg dry matter (DM), respectively) contents than those reared on vegetable waste (371 and 96 g/kg DM, respectively), chicken feed (336 and 100 g/kg DM, respectively), and restaurant waste (386 and 27 g/kg DM, respectively). Fatty acid composition was characterized by high contents of C12:0 in all experimental groups. According to the authors of that study, BSFL can represent an interesting alternative protein source in animal feeding, due to the high-quality standards of prepupae raised on different substrates. However, the growth substrate would influence the EE and ash contents. The calcium concentration in the BSFL meal was also highly variable and dependent on the larvae growth substrate [16]. Conversely, the phosphorus concentration in BSFL meal was less affected by the substrate on which they were grown [12,16].

However, Joanas-Levi and Martinez (2017) [77] observed that the nitrogen-to-protein conversion factor in insects is less than 6.25 and should instead be 4.74, 4.75, or 5.41, due to the presence of non-protein and indigestible nitrogen in the chitin of the exoskeleton [2,77]. Since the fibrous fraction of larvae is found in chitin, the major component polymer of the larval exoskeleton which is not digestible by the endogenous enzymes of monogastric animals [78], even the N encapsulated in chitin would not be digestible in the pig intestine. Other studies reported that insects contain non-protein nitrogen, such as chitin, nucleic acids, ammonia, nitrite, etc., which could lead to an overestimation of the protein content of insects [79,80]. Furthermore, the different methods used to defat (Figure 1) [2] and dry insect larvae could be responsible for differences in the standardized ileal digestibility of the AAs [81]. Indeed, it has been reported that the use of cold pressing to defat insect larvae minimally affects its apparent ileal digestibility in broilers [82]. Huang et al. (2018) [83] reported that the in vitro digestibility of AAs was higher when the method used for drying BSFL meal was the conventional one (60 °C until constant weight), compared to that with microwave irradiation.

Other scientific evidence concerns the chitin contained in the cuticle of insects. Even though it is an indigestible fiber, it has been shown that it can have positive effects on immunity [84,85]. Species and developmental stages influence the composition and quantity of chitin in insects [2]. Larvae have the lowest chitin content compared to other forms of development [2]. Chitin is a linear polymer of β-(1-4) N-acetyl-D-glucosamine units, which is found in a complex structure with cuticular proteins, lipids, and other substances [86]. Chitin can perform a bacteriostatic function and indeed, in a study conducted on piglets, it was highlighted that the use of chitin derivatives (such as chitosan) in diets is potentially capable of reducing or inhibiting the growth of pathogenic microorganisms, that cause post-weaning diarrhea [87].

The chemical and nutritional composition of BSFL and *Tenebrio molitor* larvae compared to conventional SBM is presented in Table 3.

### 3.2. Safety Issues

Safety issues limit the use of insect larvae as a feed ingredient in animal feeding. The Food and Agriculture Organization (FAO) of the United Nations establishes safety requirements for BSFL to be used in diets of livestock and pets [88]. Currently, food safety regulations constitute the main obstacles to the large-scale use of BSFL in animal feed [13]. There are also restrictive indications on the growth substrate of BSFL larvae and meat, manure, “restaurant waste”, and “other waste” are explicitly prohibited [89].

Indeed, although insects are a potential source of high-quality and quantitative proteins, their use in animal feed faces safety problems, as they could convey toxic substances produced by their defensive glands [90,91]. Benzoquinone is among the toxic substances found in *T. molitor*, which can interfere with cellular respiration, triggering kidney damage, as well as being carcinogenic in humans and animals [2]. Benzoquinone, being continuously accumulated in *T. molitor*, increases its concentration with age [2]. As reported by Hong et al. (2020) [2], it has not yet been clearly established how much benzoquinone remains in *T. molitor* larvae after the cleaning, drying, heating, and grinding processes, and what the tolerance limits of benzoquinone are in monogastric animals. Therefore, as indicated by Hong et al. [2], it is essential to indicate a control method to monitor the residual quantity of benzoquinone in products based on *T. molitor* larvae, in order to establish the level of toxicity. Currently, there are no standardized and unified production and processing procedures but only small-scale equipment with a low yield and efficiency [3].

Moreover, it has been reported that insects can express antibiotic resistance genes [92], which would mean that they can be contaminated with pathogenic microorganisms or contain mycotoxins, resulting from contaminated growth substrates [2]. However, it has been observed [93] that monitoring the presence of pathogenic microorganisms (*Escherichia coli* and *Salmonella* spp.) in the growth substrate as well as in the larvae would be an effective prevention method against the survival of such pathogens, both in larvae and in adults of *T. molitor*. Furthermore, several authors have reported that *T. molitor* larvae fed diets contaminated with different types of mycotoxins grew normally without any accumulation in their body, being able to degrade the mycotoxins [94,95]. As suggested by Hong et al. [2], it would be necessary to investigate the mechanism of resistance to mycotoxins in *T. molitor*. Another problem regarding the food safety of insects is that of the accumulation of heavy metals, deriving both from the environment and from the growth substrate [2]. A check in this regard by means of X-ray fluorescence spectrometry would also be necessary [2].

However, the introduction of BSFL meal into the diets of salmonids, trout, tilapia, and poultry (considering chickens, ducks, turkeys, and geese) has been approved and regulated by the American Association of Feed Control Officials [96].

### 3.3. Edible Insects in Studies of Swine

As already highlighted by authors who reviewed the literature on the topic, studies on pigs are more limited than those on chickens, since feed consumption is greater in pigs than in chickens, and given the high production cost and low availability of *T. molitor* and *H. illucens* larvae.

*H. illucens* larvae used as an ingredient in complete chicken diets affected their growth performance, nutrient digestibility, and blood analysis [97,98]. Other studies have indicated a clear effect of the dietary use of *H. illucens* larvae on the intestinal microbiota [97,99] and on the microbial metabolites found in the cecal digesta of laying hens or broilers [97,99,100]. It was observed that insect meals derived from *Tenebrio molitor* and *Hermetia illucens* are useful in providing apparently metabolizable energy and digestible amino acids in broiler diets [63].

BSFL (whole or partially or completely defatted) have been used in pig diets without compromising growth performance, feed intake, the digestive utilization of nutrients, or intestinal morphological characteristics [12,74,100,101,102].

Hong et al. (2020) [2], in reviewing the use of *T. molitor* larvae in diets fed to monogastric animals, concluded that up to 6% in weaning pigs’ diets and 10% in those of growing pigs, could be used as a protein source, without negative effects or with improved growth performances, as well as AA digestibility, compared to conventional protein sources.

Yu et al. (2019) [100] studied the effects of using *Hermetia illucens* larvae meal in the diet of crossbred fattening pigs (Duroc × Landrace × Large White), with inclusion levels of 4 (group H1) and 8% (group H2) compared to a diet of a control group without larvae, on growth performance, the microbiota, metabolites, and intestinal barrier gene expression in the colon. The H1 diet increased the average daily gain of pigs (0.89, 0.98, and 0.86 kg/d in the control, H1, and H2 group, respectively) and decreased the feed conversion ratio (F:G) (3.21, 2.85, and 3.23 in the control, H1, and H2 group, respectively) compared with control and H2 diets. However, there were no difference in the average daily feed intake between the control group (2.83 kg/d), the H1 group (2.77 kg/d), and the H2 group (2.87 kg/d). The H1 and H2 diets affected colon microbial population, increasing the numbers of *Lactobacillus* and different butyrate-producing bacteria (*Pseudobutyrivibrio*, *Roseburia*, and *Faecalibacterium*), and decreasing the abundance of *Streptococcus*. Moreover, diets including *H. illucens* larvae increased the number of *Clostridium* cluster XIVa bacteria. Microbial fermentation metabolites were also influenced by dietary treatments, with concentrations of total short chain fatty acids, butyrate, and isobutyrate greater in the H1 group than in the control group, and concentrations of protein fermentation products, that is, total amines like cadaverine, tryptamine, phenol, p-cresol, and skatole, lower in the H1 diet compared with the control group. The H2 diet also showed increased concentrations of butyrate and decreased concentrations of phenol, p-cresol, and skatole compared with the control group. Changes in bacterial composition and in their metabolites were associated with changes in gene expression in the colonic mucosa. Regarding the immune status of the intestinal mucosa, pigs in the H1 group showed a more reduced expression of TLR-4 and proinflammatory cytokines (IFN-γ) compared to pigs in the control group, and upregulated anti-inflammatory cytokine (IL-10) and intestinal barrier genes (ZO-1, occludin and mucin-1). In the pigs of the H2 group, there was an increased expression of ZO-1 compared to the control group. According to the results of this study, the inclusion of *Hermetia illucens* larvae in pig diets can improve the immune status of the intestinal mucosa of pigs, through an alteration of the bacterial composition and its metabolites. The findings provide a new perspective on the use of this insect’s larvae as a sustainable protein source rich in nutritional ingredients for pigs.

Yu et al. (2019) [103] also evaluated the effects of including different percentages of *Hermetia illucens* larval meal (0, 4, and 8%; named as groups HI0, HI4, and HI8, respectively) in the diet of crossbred female finishing pigs (Duroc × Landrace × Large White) on their growth performance, carcass traits, and meat quality, including fatty acid composition. The effects of the administration of *Hermetia illucens* larvae on the relative mRNA expression of genes related to lipid metabolism and to myosin heavy-chain (MyHC) in *longissimus thoracis* (LT) muscle of the finishing pigs were also evaluated. The HI4 group showed a higher final body weight and average daily gain as well as a lower feed/gain ratio compared to the HI0 and HI8 groups. There were no significant differences for average daily feed intake among groups. Varying dietary *H. illucens* larvae meal inclusion did not affect the 45 min and 24 h pH values, the 45 min and 24 h L*, a*, and b* parameters, drip loss, or shear force. Groups HI4 and HI8 had a greater loin area, marbling scores, and inosine monophosphate (IMP) content in the LT muscle compared to group HI0. The HI4 group had a higher intramuscular fat content compared to the HI0 group. In addition, HI4 group showed a higher intramuscular fat content in the LT muscle than the HI0 group. Although dietary treatments influenced the concentrations of several individual fatty acids in the LT muscle of pigs, the total saturated (SFA), monounsaturated (MUFA), polyunsaturated (PUFA) fatty acids, and n-6 PUFA/n-3 PUFA ratio did not differ significantly between the three experimental groups. The expression level of fatty acid synthase (FAS) mRNA was significantly increased in the HI4 and HI8 groups compared to the HI0 group. Furthermore, the mRNA expression degree of acetyl CoA carboxylase α (ACCα) and lipoprotein lipase (LPL) was also increased in the HI4 group compared to the HI0 group. As regards genes related to the LT muscle fiber composition, there was an increased mRNA expression level of myosin heavy-chain (MyHC)-IIa with the HI4 diet compare to HI0. According to that study, the inclusion of H. illucens larvae in the diet has a positive influence on growth performance and meat quality, and the authors argue that the underlying mechanism could be related to the alteration of the lipogenic potential induced by *H. illucens* larvae.

Altman et al. (2019) [76] conducted a very interesting and multifaceted study in which they studied the physico-chemical and sensory traits of pork from barrows ((Pietrain × (Large White × Landrace)) fed diets containing *Spirulina* (*Arthrospira platensis*) or black soldier fly (*Hermetia illucens*) partly defatted larval meal as alternative protein ingredients to soybean meal. Pork quality was evaluated under highly oxygenated modified atmosphere industrial packaging conditions. Diets were administered during three growth periods (25–50, 51–75, and >75 kg), and two of the three were experimental diets as both in the first and second growth periods, 50 (replicate 1) or 75% (replicate 2) of the soybean meal was replaced with Spirulina or larval meal, while in the last fattening period, 100% of the soybean meal was replaced. The third diet was administered to the control group animals and was a typical diet in which the primary protein source was soybean meal. The diets, as a consequence of the replacement of soybean meal, differed markedly in the integration of essential amino acids. Overall, the dietary protein source rarely affected the physico-chemical parameters of pork (Table 4) [76], even when packaged in standardized industrial conditions.

The experimental diets provided products that hardly differed in terms of sensory aspects from those from the control group, and indeed, the differences found were interpreted as sensory improvements, such as the more intense smell and greater juiciness. The two alternative protein sources influenced the fatty acid composition of backfat, showing a higher polyunsaturated fatty acid content compared to using soybean meal as the primary protein source. Furthermore, the lauric acid (C12:0) content of backfat was higher (five times) in the group fed *Hermetia illucens*, and the authors suggest that this fatty acid may be a biomarker for pork from animals fed this alternative protein source.

Spranghers et al. (2018) [74] conducted two studies to evaluate the influence of different amounts of prepared fat from BSF prepupae on the intestinal microbiota of pigs, simulating digestion in the small intestine of piglets by means of the in vitro technique, but also by conducting an in vivo test in weaned piglets. An incubation medium was prepared containing a synthetic diet, a microbial inoculum from a donor piglet, and 0.20, 0.50, 1.00, and 1.50 g/100 mL of BSF fat medium. At the end of the incubations (37 °C for 4 h), several aliquots of medium were taken and coliforms, D-streptococci, lactobacilli, and total anaerobic bacteria were counted. Weaned piglets (fifty-six, males and females, weaned on 21 days of age; 6.178 ± 0.562 kg) were fed diets containing whole (4 and 8%) and defatted (5.4%) BSF prepupae, compared with a control diet containing soybean as a protein and fat source. The experiment lasted 15 days. The average daily gain (ADG), average daily feed intake (ADFI), and feed to gain ratio (F:G) were registered. After slaughter, the digesta and sections of the intestine were collected. The total ileal and fecal apparent nutrient digestibility (gross energy, dry matter (DM), CP, and ether extract (EE) were calculated. The C12:0 content in the BSF prepupae was 57.9 g/100 g of ether extract. From the in vitro digestibility study, it emerged that at the inclusion level of 1.00 g/100 mL (corresponding to 0.58 g C12:0/100 mL), the growth of lactobacilli was suppressed, with a more marked effect against D-streptococci. The highest inclusion level of prepupal fat (1.50 g fat/100 mL, corresponding to 0.87 g C12: 0/100 mL) resulted in approximately 2-fold log reductions in D-streptococci. From the in vivo trial, piglets fed diets containing BSF showed log reductions of only 0.5-fold for D-streptococci in their intestines. There were no differences between the experimental groups regarding ADG, ADFI, and F:G. The apparent fecal digestibility of nutrients did not differ between groups. The ileal protein digestibility of the 8% full-fat BSF-containing diet was lower (67.4%) than that of the control (69.7%), while the observed values for the 4% full-fat BSF and defatted BSF diets were greater (73.3%). The authors conclude that considerable amounts of full-fat or defatted BSF (up to 8%) may be used in piglet diets to replace soybean products, without having negative effects on growth performance.

Chia et al. (2021) [102] studied the effects of substituting fish meal (FM) with full-fat black soldier fly larval meal (BSFLM), according to replacement rates (*w*/*w*) of 25 (D25), 50 (D50), 75 (D75), or 100% (D100), in the diets of pigs (hybrid Large White and Landrace) slaughtered at weights of around 100 kg. The dietary treatment influenced growth performance, with greater average daily gains in the D50, D75, and D100 groups compared to the D0 one. Final body weights were greater in the D50 and D100 pig groups than in the D0 and D25 ones, while feed conversion ratios were lower in D50, D75, and D100 finisher pigs, compared to the D0 and D25 groups. The carcass yield of pigs fed diets containing BSFLM with FM replacement rates of 50, 75, or 100% was greater than for pigs from the control group, consuming 100% FM as protein source. The crude protein content of the different tissues analyzed was high, varying (on a dry matter basis) between 65 (in the heart) and 93% (in the lung) among all dietary treatments. Furthermore, the different tissues of pigs fed diets containing 50–100% BSFLM had a higher crude fat content than those of pigs fed diets prepared with a 0–25% replacement rate of FM with BSFLM. According to the authors, the improvement in growth performance of pigs fed with increasing levels of BSFLM as a replacement for FM in the diet is indicative of the improved palatability of the diet together with a sufficient consumption of digestible nutrients. The reduction in the feed conversion ratio with the greater levels of replacement of FM with BSFLM opens up the prospect of a reduction in feed costs for pig production.

Crosbie et al. (2020) [12] conducted two experiments on growing barrows (Yorkshire × Landrace × Duroc; 25.1 kg BW ± 0.41 kg) in order to determine the standardized ileal digestibility (SID) of amino acids (AAs) (Exp. 1) and net energy (Exp. 2) of two samples of black soldier fly larvae meal (BSFLM), one full-fat (FF; 42.5% crude protein, CP, as-fed) and one defatted (DF; 40.8% CP, as-fed). For this purpose, two corn starch-based diets were formulated, containing FF (50%, as-fed basis) or DF (36.5%, as-fed basis) BSFLM as unique dietary AA sources. The study showed that the SID of CP (i.e., 80.6%; average for FF and DF BSFLM) and Lysine (i.e., 88%; average for FF and DF BSFLM) did not vary between FF and DF BSFLM. The SIDs of some AAs (i.e., Arg, Val, Ala, and Pro) were or tended to be lower for the FF than for DF BSFLM, while the opposite was the case for the SID of Met. According to the authors, this was most likely attributable to the relative major concentration of NDF-N in FF BSFLM. Furthermore, the authors report that the differences in the SID of the AAs is due to the methods applied to defat and/or dry the BSFLM. The digestible energy (4927 vs. 3941 ± 75 kcal/kg), the metabolizable energy (4569 vs. 3396 ± 102 kcal/kg), and the calculated net energy (3477 vs. 2640 ± 30 kcal/kg; 3479 vs. 2287 ± 28 kcal/kg, using the Noblet or Blok equations, respectively) were higher for FF than for DF BSFLM. Furthermore, the apparent total tract digestibility of neutral detergent fiber and acidic detergent fiber was higher for FF than for DF BSFLM. The study therefore revealed that both FF and DF BSFLMD had high SID values for the majority of AAs, although FF BSFLM provided greater net energy for growing pigs. According to the authors of the study, both FF and DF BSFLM are potential alternative protein sources in the formulation of diets for growing pigs.

Ipema et al. (2021) [104] evaluated the opportunity of providing live BSFL larvae as an environmental enrichment to benefit pig welfare, as tested in broiler chickens.

The effects of insect larvae meal supplementation in pig diets are presented in Table 5.

## 4. Rapeseed Meal and Legume Grains

### 4.1. Nutritional Value

Rapeseed meal (RSM) is a by-product from oil and biofuel production [29]. RSM, with a crude protein content which varies between 33.7 and 35.6% (as fed) [105], can represent an opportunity to diversify the ingredients of pig diets using home-produced feed [29]. However, the high content of fiber and anti-nutritional factors such as glucosinolates, sinapine, tannins, and erucic acid have limited the use of rapeseed meal in pig diets over time [106,107]. Glucosinolates are sulfur-containing glycosidic compounds whose decomposition produces products capable of reducing feed intake, altering the production of thyroid hormones [107], and consequently influencing metabolism and animal performance. However, the production of hormones by the thyroid gland can be inhibited depending on the nature and concentration of degradation products from RSM glucosinolates [107]. An increase in weight of the thyroid gland was observed in pigs fed RSM, despite a level of total glucosinolates lower than the recommended limit of 2.1 mmol/kg [29]. Phenolic compounds such as tannins in RSM can also reduce protein digestibility and interfere with protein metabolism [108]. A higher thyroid gland weight but normal thyroid hormone concentration was also observed in pigs fed 6–10% RSM in the growing-finishing phase [109]. However, traditional rapeseed varieties were selected in order to decrease the levels of erucic acid in the oil and glucosinolates in the non-oily part of the seed, as well as to obtain an improved AA profile [105]. Indeed, due to progress in plant breeding, rapeseed seeds containing low levels of erucic acid (<2%) in the oil and glucosinolates (<15 µmol/g) in defatted meal have recently become available on the market, called canola in North America, and “double-zero” or “double-low” rapeseed or 00 rapeseed in Europe [105].

Grain legume seeds, such as fava beans, peas, lupins, and chickpea, contain protein, soluble and insoluble fiber, slowly digested starch, micro- and macronutrients, vitamins, and numerous bioactive phytochemicals, such as flavonoids and other antioxidants [31]. The CP content of common grain legumes varies from 20 to 30%, with the highest content in yellow lupin (324–381 g/kg dry matter), but they are low in sulfur-containing amino acids (methionine and cystine) and, in addition, in tryptophan when compared to SBM [31]. Moreover, the use of legumes in animal diets has been limited due to the presence of secondary plant metabolites, so-called anti-nutritional factors (ANFs). ANFs include condensed tannins (proanthocyanidins, non-hydrolysable), protease inhibitors (trypsin and chymotrypsin inhibitors in most legume species), alkaloids (toxic amines mainly contained in lupins), lectins (phytohaemagglutinins, glycoprotein compounds mainly in common beans), pyrimidine glycosides (vicine and convicine in fava beans), and saponins (glycosides contained in many plants) [31]. These secondary plant compounds reduce palatability (tannins and alkaloids), nutrient digestibility (tannins, protease inhibitors, lectins), or may have toxic effects (alkaloids) [24,31]. Furthermore, negative effects on pigs, such as excessive fermentation, flatulence, and diarrhea, may be caused by galactosides contained in high percentages in some grain legumes [31]. Moreover, in many legumes such as lupins, non-starch polysaccharides (NSPs) are present in considerable quantities, which can have negative effects on pigs by reducing the passage rate of the digesta and feed intake and growth performance [31]. The high neutral detergent fiber content of legume seed hulls has also been reported to reduce nutrient digestibility in pigs [24,108]. The authors also suggested that the complex structure and conformation of legume seed proteins is, at least partially, responsible for their reduced digestibility, as it makes them more resistant to proteolysis [24,31]. However, advances in plant breeding have enabled the commercial release of cultivars with improved nutritional values, as well as lower contents of secondary metabolites [31]. Moreover, the use of different processing methods can reduce or eliminate ANFs, such as physical treatments (e.g., decortication and soaking), thermal treatments (e.g., extrusion and cooking) or biological methods (e.g., germination and enzyme integration) [31,110].

The chemical and nutritional composition of pea, yellow lupin, and RSM compared to conventional SBM is presented in Table 6.

### 4.2. The Use of Rapeseed Meal and Legume Grains in Pig Diets

Batterham et al. (1993) [111] investigated the tolerance of pigs in the growth phase (between 20 and 50 kg) to trypsin and chymotrypsin inhibitors contained in chickpeas (Cicer arietinum) and pigeon peas (Cajanus cajan). Gradually increasing levels of meals rich in these inhibitors were added to the diets of growing pigs, in order to evaluate their influence on growth performance and internal organs. Diets containing either 250, 500, or 750 g kg^−1^ of Opal chickpea, dehulled Tyson chickpea, or dehulled pigeon pea meals were fed to pigs and compared to a wheat-soyabean meal control diet. Pigs that consumed the two chickpea meals showed growth performance comparable to that of the pigs fed the control diet based on soybean meal. Conversely, the pigeon pea meal-based diet decreased the growth rate and feed intake, while it increased the feed conversion ratio. Moreover, the chickpea meals had no influence on organ weights at the inclusion levels used, while the pigeon pea meal influenced liver and pancreas weights, suggesting the presence of other anti-nutritional factors. The authors concluded that growing pigs would have a dietary tolerance of at least 4.7 and 4.5 mg/g of trypsin and chymotrypsin inhibitors, respectively, amounts that would occur with the inclusion of 750 g kg^−1^ of chickpea meals in the diet. According to the authors, it is not likely that these threshold levels would be exceeded in common diets that include most legume seeds. According to the results of the study, the dehulled pigeon pea meal contains other anti-nutritional factors for growing pigs.

Contrary to Batterham et al. (1993), a maximum dietary tolerance level of approximately 0.5 mg trypsin inhibitor/g has been reported for fattening pigs [31].

Jansmann et al. (1993) [112] evaluated the effects of diets prepared with different field bean cultivars (300 g/kg) in piglets. Colored-flowered field bean cultivars (1.0–2.3 g condensed tannin/kg diet) showed a significantly lower ileal digestibility of CP and most AAs than those without white-flowered tannin. Flis et al. (1999) [113] did not observe negative effects on the growth performance of pigs (25–63 kg body weight) fed a fava bean diet having 0.59 g/kg of condensed tannins, compared to pigs fed diets that contained 0.07 g/kg.

Mustafa et al. (2000) [24] conducted a study in which they compared different diets for crossbred pigs, based on barley and wheat supplemented with SBM (control), different types of chickpeas (Kabuli and Desi, 300 g/kg), or field peas (300/g kg). There were lower dry matter and gross energy digestibility coefficients in pigs fed the Desi (Indian origin) chickpea-supplemented diets than in pigs fed the Kabuli (Mediterranean origin) chickpea-supplemented diets. However, the dry matter and gross energy digestibility for both chickpea-supplemented diets (i.e., Desi and Kabuli types) were comparable to those of soybean- and pea-supplemented diets. Indeed, the two varieties of chickpeas differed in their nutritional composition, with the Kabuli type containing less fiber, more starch, and more fat compared to the Desi type. The authors hypothesized that the lower growth performance (i.e., daily gain and feed conversion) of growing pigs fed diets supplemented with chickpeas compared to those fed SBM or peas could be related to the higher fiber contents of the chickpea-based diets. Indeed, according to the authors, the inclusion levels of the two types of chickpeas in the pigs’ diets were not such as to exceed the tolerance levels of trypsin and chymotrypsin inhibitors indicated in the work of Batterham et al. (1993) [111]. The crude protein digestibility did not differ for both diets containing the two types of chickpeas and those supplemented with peas, but was 9.6% lower than that of the SBM-supplemented diet. However, during the finishing period and throughout the experiment, the dietary treatments did not affect pig performance, and indeed, there were no significant differences in carcass traits among pigs fed diets supplemented with SBM, peas, or chickpeas.

Several studies have reported that grain legume processing effectively improves starch and protein digestibility in pigs, thanks, at least in part, to a reduction in secondary plant metabolites. The inclusion of hulled lupins in pig diets improves their nutritional value by increasing the feed intake and feed conversion ratio compared to whole seeds [114]. The extrusion of peas at a temperature of approximately 115 °C increases the apparent (AID) and standardized ileal digestibility (SID) of CP and AAs, as well as starch digestibility and energy supply in growing pigs [115]. In the same manner, improved AID and SID of CP and most AAs are reported when extruded peas (135 °C) are fed to weaned piglets [116]. Furthermore, the inclusion of extruded peas (130 °C for 30 s) in diets of growing-finishing pigs increase growth and the feed conversion ratio compared to untreated peas [117]. A lupine-based diet supplemented with the enzyme α-galactosidase increases the digestibility of α-galactosides, AAs, energy supply, N retention, and growth performance in growing pigs [118].

Christodoulou et al. (2006) [119] evaluated the effect of replacing soybean meal with extruded chickpeas on meat quality in the grower and finisher pig diets. There were small differences in the meat’s chemical composition among experimental treatments, i.e., control (soybean-based diet) and the experimental groups fed diets containing 100, 200, and 300 kg/t of extruded chickpeas. Similarly, the meat’s fatty acid composition, pH, and color did not differ between treatments, although the control group showed a lower cooking loss. As regards sensory evaluation, slightly greater scores for tenderness and juiciness were attributed to the control group than the chickpea treatments. The authors concluded that the replacement of soybean meal with extruded chickpeas, up to inclusion levels of 300 kg/t in isoenergetic and isoprotein diets for growing-finishing pigs, does not affect meat quality.

Jezierny et al. (2010) [31], in their review on grain legumes as a protein source in pig nutrition, reported that fava beans may be used in growing and finishing pig diets up to 150 and 250 g/kg, respectively, preferring white-flowered cultivars for their low tannin content. An inclusion level of up to 400 g/kg of peas in diets is recommended for growing and finishing pigs, preferring, even for peas, the white-flowered cultivars due to the low tannin content compared to the flowered ones. The recommended inclusion level for lupins in diets for growing and finishing pigs is 200 g/kg, but only up to 150 g/kg for *L. albus* in diets for growing pigs (30–60 kg body weight).

A recent work by Grabež et al. (2020) [5] addresses the effect of replacing SBM with rapeseed meal and fava beans (RSM/FB) on growth performance, carcass quality, metabolite status, and meat quality in Norwegian crossbred ([Landrace × Yorkshire] × Duroc) growing-finishing pigs. Pigs fed the RSM/FB diet did not show different growth performance compared to pigs fed the SBM diet during the entire test period, except for a higher feed conversion ratio (F:G, kg/kg) during the finishing phase (2.44 vs. 2.33 for RSM/FB and SBM pig diets, respectively). According to the authors, the higher F:G in the finishing period could be related to anti-nutritional factors present in both RSM and FB. Both RSM and FB are characterized by a higher fiber content than SBM and also contain several anti-nutritional factors responsible for reduced feed use. However, carcass traits were not affected by diet [5], in agreement with other authors who had examined the effect of administering legume and rapeseed cakes to pigs [33,34,120]. However, Grabež et al. 2020 [5] reported that pigs fed RSM/FB as a replacement for SBM exhibited darker meat, that is, lower L* (lightness) and b* (yellowness) parameters. However, darker meat, i.e., with lightness values (L*) lower than 48, can have higher visual ratings without compromising meat quality [5,120]. Regarding meat fatty acid composition, RSM/FB affected the contents of individual fatty acids but not the total of SFA, MUFA, and PUFA. Previously, it has been reported that the complete replacement of SBM with RSM/FB, together with a reduced use of tallow in a balanced diet for digestible energy, does not influence the total SFA and MUFA contents of pork muscle, although it tends to reduce PUFA [121]. Replacing SBM with RSM/FB in growing-finishing pigs affected metabolites extracted from pre-rigor Longissimus thoracis muscle and meat after a chilled storage period (7 days at 4 °C). The metabolite profile of pre-rigor muscle showed reduced levels of β-alanine and glucose when pigs were fed RSM/FB. Moreover, the reduction of oxidized metabolites (glycine and pyroglutamic acid) in the muscle of the RSM/FB pigs would indicate less oxidative stress in the muscle. Furthermore, the decrease in pyroglutamic acid would suggest the efficient synthesis of glutathione and an improvement in the cell’s defense mechanisms [5]. The results were in agreement with those of Skugor et al. (2019) [29], who highlighted an increased regulation of genes active against oxidation and reactive oxygen species (ROS) in RSM-fed pigs. Grabež et al. [5] concluded that the aromatic characteristics of pork from pigs fed the RSM/FB diet were desirable, the concentrations of free amino acids and metabolites characterized by a sweet flavor were increased, while at the same time, the reheated flavor was reduced. The results of that study therefore show a desirable quality of pork obtained from a diet containing rapeseed meal and fava beans.

Previous studies [33,34] observed that pigs fed a diet containing RSM and field beans as a replacement for SBM provided less tasty but more tender pork than pigs fed an SBM-based diet.

A meta-analysis conducted by Hansen et al. (2020) [122] showed that up to 30% rapeseed meal in growing-finishing pig diets did not compromise growth performance compared to an SBM-based diet, when added to a nutritionally balanced diet.

Skugor et al. (2019) [29] studied the effects of including 20% RSM in diets for growing-finishing pigs, as a replacement for SBM, on growth performance, carcass, and meat quality characteristics. Furthermore, the authors aimed at investigating the diet’s influence on gene expression in Longissimus dorsi muscle and identifying the major factors responsible for phenotypic differences. The inclusion of 20% commercial expeller pressed RSM in pig diets reduced the growth performance and dressing percentage of growing-finishing pigs compared to an SBM control diet. However, the meat quality characteristics were not affected by dietary treatment. According to the authors, changes in the gene expression of skeletal muscle from pigs fed the RSM diet were likely due to an increased amount of fiber and polyunsaturated fatty acids, as well as bioactive compounds, such as glucosinolates. In accordance with a lower growth performance, the negative action of growth regulators (IER5, KLF10, BTG2, KLF11, RETREG1, and PRUNE2) was observed in pigs of the RSM group. Furthermore, the increased expression of different muscle genes (PDK4, UCP3, ESRRG, and ESRRB) implicated in glucose and lipid metabolism and mitochondrial function in pigs fed RSM was detected, suggesting a lower availability of energy and nutrients in RSM-fed pigs and the intervention of well-known metabolic controllers to ensure energy homeostasis. Regarding genes regulating protein metabolism, several genes were implicated in more pronounced proteolysis (ABTB1, OTUD1, PADI2, and SPP1) and reduced protein synthesis (THBS1, HSF4, and AP1S2) in the muscle tissue of RSM pigs. Moreover, the authors observed an increased expression of genes regulating lipolysis, fatty acid oxidation (greater levels of NR4A3, PDK4, and FGF21, and a drop in adropin, ELOVL6, and CIDEC/FSP27), and oxidative stress (GPX1, GPX2, and TXNIP) in the muscle of pigs fed RSM. The study of gene expression deepens the knowledge of the molecular mechanisms underlying phenotypic observations.

Chen et al. (2018) [30] carried out a 3-week study in young pigs (17.8 ± 2.7 kg initial BW) to compare a soybean meal-based control diet with a rapeseed-based diet (200 g/kg on an as-fed basis). The metabolic effects of the two diets were examined by analyzing digesta, liver, and serum samples from these animals. A reduced apparent ileal digestibility of AAs was observed in pigs that consumed the RBM diet. However, although the RSM diet had a higher fiber content, the microbial fermentation products in digesta were not affected, i.e., short-chain fatty acids and secondary bile acids. Increased contents of oxidized metabolites (e.g., oxidized glutathione) and aldehydes, and decreased levels of ascorbic acid and lipids containing docosahexaenoic acid were found in the liver and serum of RSM-fed pigs, highlighting an alteration of the redox balance in these young pigs. Contrary to what was observed by Grabež et al. [5], Chen et al. [30] reported that the metabolic markers of oxidative stress (pyroglutamic acid and butanal) were more expressed in the liver of pigs fed the RSM diet. Processing interventions are recommended in order to increase the use of rapeseed feed ingredients in pig diets.

Zmudzińska et al. (2020) [4] evaluated the effect of a total dietary replacement of SBM with legume grains (pea and yellow lupin) and RSM on the growth performance and meat quality of both sexes in DanBred hybrid piglets. There was no interaction between sex and diet. Replacing SBM with legume grains and RSM in growing-finishing pig diets reduced the final body weights. Indeed, the dietary treatment decreased the daily weight gain in the period between 35 and 83 days, and throughout the whole rearing period (0–83 days). The experimental factors did not influence most of the meat quality characteristics. Pigs fed legume grains and RSM showed lower fatness than those fed SBM. The authors concluded that including peas, yellow lupins, and RSM in pig diets does not affect meat quality, although growth performance may be impaired.

He et al. (2023) [28] conducted a study with the aim of determining the effects of replacing SBM with different plant protein sources (rapeseed meal, cottonseed meal, and sunflower seed meal) on the growth, apparent nutrient digestibility, serum parameters, including free amino acids, and the intestinal microbiota of growing pigs (Duroc × Landrace × Yorkshire) weighing 50 to 75 kg. The three-meal mixture was added in one of the three experimental groups (corn-oybean–various meal group, CSM) in a 1:1:1 ratio and as a partial replacement of 10.99% of SBM compared to the control group (corn–soybean meal group, CON). Pigs in the CM group (corn–various meal group, CM) received a diet in which the three-meal mixture (7.69% rapeseed meal, 7.69% cottonseed meal, and 7.68% sunflower seed meal) totally replaced SBM. The dietary treatments did not influence the average daily gain, average daily feed intake, or feed-to-gain ratio of growing pigs weighing between 50 and 75 kg. The same results were obtained for the crude protein, crude fat, and gross energy digestibility of the three experimental diets. As regards serum parameters, the CM group showed increased values of alanine aminotransferase (ALT) and triglyceride (TG), but reduced urea values. Serum free amino acids were not affected by the dietary treatment. Regarding the composition and diversity of fecal microbiota, the CM group showed a decrease in Euryachaeota abundance at the phylum level compared to the CON group, suggesting improved intestinal crude fiber-digesting bacterial flora. The results are indicative of the useful combination of miscellaneous meals (rapeseed meal, cottonseed meal, and sunflower seed meal) as potential alternative feed ingredients to SBM in swine diets.

The effects of the use of RSM and/or legume grains in pig diets are summarized in Table 7.

## 5. Conclusions

Microalgae are currently relatively expensive to produce on a large scale compared to other animal feeds. Research focuses on their use as diet supplements, primarily to influence gut health. When used as protein ingredients in post-weaning piglet diets, at an inclusion level of up to 10%, growth performances are decreased, even if the meat quality is not negatively affected. This decrease in pig growth performance is linked to the gelation and therefore the low digestibility of Spirulina proteins in the gut, due to the resistance of their cell wall carbohydrates to digestion. Furthermore, other factors that contribute to the limited use of Spirulina in animal feed are palatability, dried powdery form, and odor.

From the literature consulted, it emerges that insect larvae, i.e., *Hermetia illucens* and *Tenebrio molitor*, can be used in growing pig diets up to an inclusion level of 10%, without negative effects on growth performance and nutrient digestibility. Therefore, the results confirm insect larvae as potential sources of sustainable protein in pig feeding. However, for their large-scale use, the problems of safety, high non-competitive production costs, as well as acceptability by consumers should be addressed. Studies, similarly to those on laying hens or broiler chickens, are exploring their potential added value (as a feed additive and not an alternative protein source) in pig feeding, i.e., that of regulating intestinal microbiota and microbial metabolism products, probably through the effects of chitin, lauric acid, and/or antimicrobial peptides, especially during the post-weaning period.

Locally produced protein sources, such as rapeseed meal and grain legumes, used in combination with cereals and taking care not to exceed the anti-nutritional factor threshold values, can be included in diets of growing-finishing pigs, without any negative effect on growth performance and meat quality. The use of these ingredients can contribute to the greater sustainability of pig farming in Europe, as well as a reduction in feeding costs. Furthermore, the organic farming sector, which is in general GMO-free, would particularly benefit from these alternative protein sources, given that the majority of soybean imported from American is genetically modified.

## Figures and Tables

**Table 1:** Chemical and nutritional composition of *Spirulina platensis* compared to conventional soybean meal (SBM) (% or otherwise indicated, as-fed basis).

Proximate Composition	Amount	Data Range	Amount	SBM
Dry matter	93.8	91–96	93.5	89
Crude protein	60.1	60–70	63.78	43.8
Crude fat	6.84	4–16	4.73	1.5
Crude fiber		3–7		
Carbohydrates (total)		14–19		
Neutral detergent fiber				13.3
Acid detergent fiber				9.4
Ash	6.70	3–11	9.02	
Energy (kJ/100 g)		1504.0		
Amino acid composition				
Alanine	5.21			
Arginine	4.67			3.23
Aspartate	6.50	5.20–6.00		
Cysteine	0.577			0.70
Glutamate	9.39	7.04–7.30		
Glycine	3.38			
Histidine	1.03			1.17
Isoleucine	4.15			1.99
Leucine	6.00	5.90–8.37		3.42
Lysine	3.19	2.60–4.63		2.83
Methionine	1.59	1.30–2.75		0.61
Phenylalanine	2.95	2.60–4.10		2.18
Proline	2.43			
Serine	3.46			
Threonine	3.32			1.73
Tryptophan	1.13			0.61
Tyrosine	3.00	2.60–3.42		1.69
Valine	4.66			2.06
Fatty acid composition (% total fatty acids)				
12:0	0.00			
14:0	1.01		0.17	
16:0	37.6	25.8–44.9	42.9	
16:1c9	13.5	2.3–3.8		
18:0	1.00	1.7–2.2	3.4	
18:1c9	1.47	1.01–1.66	1.6	
18:1c11	0.21		0.73	
18:2 n-6	17.0	11.1–12.0	16.5	0.69
γ-linolenic (18:3 ω-6)		17.1–40.1	23.2	
C18:3 ω-3			0.43	
20:0	0.160			
SFA	0.00		49.2	
MUFA	0.00		8.9	
PUFA			41.9	
n-6 FA			40.4	
n-3 FA			0.4	
n-6/n-3 FA			101	
Pigments, diterpene, vitamins				
Chlorophyll a ^1^ (µg/g)	1197			
Chlorophyll b ^2^ (µg/g)	45			
Total chlorophylls ^3^ (µg/g)	1242			
Total carotenoids ^4^ (µg/g)	697			
Total chlorophylls and total carotenoids ^5^ (µg/g)	1939			
β-Carotene (µg/g)	233			
Thiamine (B1) (mg/kg)		35–40		
B2 (mg/kg)		30–46		
Niacin (B3) (mg/kg)		130–150		
B6 (mg/kg)		5–8		
B12 (mg/kg)		1.5–2.0		
Foliate (mg/kg)		0.50		
α-Tocopherol (µg/g)	24.6			
β-Tocopherol (µg/g)	0.907			
γ-Tocopherol (µg/g)	0.932			
α-Tocotrienol (µg/g)	n.d.			
Minerals				
Calcium	0.12	0.12		0.32
Magnesium		0.33		0.27
Phosphate	1.30	1.30		0.65
Potassium		2.6		1.96
Sodium	0.45	2.2		0.01
References	[9]	[7] ^6^	[38]	[2,3]

FA: fatty acid, SFA: saturated fatty acid, MUFA: monounsaturated fatty acid, PUFA: polyunsaturated fatty acid, ^1^ Chlorophyll a = 11.24 × A662 nm − 2.04 × A645 nm. ^2^ Chlorophyll b = 20.13 × A645 nm − 4.19 × A662 nm. ^3^ Total chlorophylls (Ca + b) = 7.05 × A662 nm + 18.09 × A645 nm. ^4^ Total carotenoids (Cx + c) = (1000 × A470 nm − 1.90 × Ca − 63.14 × Cb)/214. ^5^ Total chlorophylls and carotenoids = (Ca + b) + (Cx + c). ^6^ % dry matter basis, n.d.: not detected.

**Table 2:** Effects of Spirulina (SP) supplementation in pig diets.

References	Phase	Breed	Sex	Ingredient Type	Supplementation Level (As-Fed Basis)	Age	Performance
[9]	Post-weaned piglets	(Large White × Landrace) × Pietrain	Male	Freeze-dried SP SP + CAZymes SP + L	10%	12.0 ± 0.89 kg (28 d, 28 d growth trial)	Decreased growth performance (final BW reduced by 9.1% on average) in pigs fed SP compared to C diet Greater TTAD of CP in C group than in the other dietary treatments Greater TTAD of CF and ADF in SP + L group than in SP and C groups No difference in meat quality No effect of SP on pork lipid oxidation after 7 d storage period L more efficient than CAZymes in SP cell wall degradation in the gut SP CP digestion in the gut still a challenge
[17]	Growing pigs	Danube White pigs	-	Dry biomass SP	2, 3 g/capita/d in addition to C diet	12.15–12.471 kg (47 d experiment period, 30.9–33.9 kg final BW)	Increased ADG in pigs fed SP with a trend in better FCR No effects on plasma biochemical parameters and blood cell composition Trend towards fewer diseased animals in SP-fed pigs
[19]	Weaning pigs in three experiments and different growth phases (d) Exp. 1, 3: 0–7 d (1) 7–14 d (2) 14–28 d (3) Exp. 2: 0–7 d (1) 7–14 d (2) 14–28 d (3) 28–42 d (4)	Exp. 1, 3: PIC, L326 × C22 Exp. 2: PIC, L42 × L19	-	SP replaced SBM in pelleted (P) or meal (M) diets, on an equal lysine basis in the C diet	Exp. 1: increasing from 2 (1), 5 (2) to 20 (3) g/kg P diet in (1) (2) M diet in (3) Exp. 2: 1 g/kg (0–42 d) or 2 g/kg in 0–7 d, 0–14 d, 0–28 d or 0–42 d (duration effect of SP supplementation) all M diets Exp. 3: 2 g/kg M or P diets (effect of form supplementation)	Exp. 1: 3.7 kg (28 d growth trial) Exp. 2: 5.6 kg (42 d growth trial) Exp. 3: 4.0 kg (28 d growth trial)	Exp. 1: no differences in pig performance in (1) and (2) phases; increased ADG in (3) for pigs fed 20 g/kg SP compared to pigs fed C diet Exp. 2: no differences in ADG or ADFI in 0–14 d; better FCR in pigs fed 1 g/kg SP in 0–42 d than in un-supplemented pigs, or fed 2 g/kg SP in 0–28 d than in pigs fed in 0–7 or 0–14 d; no differences in ADG or ADFI in 0–42 d Exp. 3: no differences in ADG, ADFI, or FCR due to dietary treatment No effects due to SP supplementation or feed processing
[38]	Gestating and lactating sows	Landrace × Large White	Gilts	SP	20 g/d in tablets	119.3 ± 8.2 kg (5.6 months)	Decreased ADG in male offspring (slaughtered at 4 months) from SP supplemented gilts (not in females) No difference in physicochemical characteristics of offspring meat Trend towards improvement of the PUFA/SFA ratio in the meat of the offspring of supplemented gilts Sex effect of maternal SP supplementation in the offspring
[54]	Weaning pigs	(Large White × Landrace) × Piétrain,	Barrows and female	SP and Chlorella (CV) spray-dried	1% both for SP and CV	9.1 kg (28–42 d)	No difference in ADFI, ADG, and FCR Reduced diarrhea in CV pigs compared to un-supplemented (NC), SP, or pigs fed diets supplemented with 0.2% colistin sulphate (PC) Increased total tract digestibility of gross energy in SP and CV pigs compared to NC and PC Increased villus height at the jejunum in SP and CV pigs compared to NC and PC Potential effects of SP and CV supplementation on mild gut disorders
[57]	Fattening pigs	Big White Polish/Polish White Zwisloucha, dams × Hampshire/Pietrain	-	*Spirulina maxima* (Sm) enriched with microelements (Fe, Cu, Zn) by biosorption	Addition in the experimental diets (starter, grower, finisher) instead of the organic salts	20.9 ± 2.2 kg (87 d feeding experiment)	No difference in ADFI, ADG, and FCR throughout the trial and in the different growth periods No difference in the balance of nitrogen retention Decreased Chol LDL (17.05%) and total Chol (9.43%) in pigs fed enriched Sm compared to C diet Increased redness (13%) and decreased drip loss (34%) values in meat from experimental group
[58]	Fattening pigs	Landrass × Yorkshire		Fresh (25% DM) SP	2 g/capita/d	85 d (95 kg final BW)	Increased ADG in pigs fed fresh SP (by 9.26% compared to C group) 100 kg BW reached 7.37 days earlier Decreased (by 1.28 MJ) AE consumed/kg weight gain in SP group pigs compared to C Greater carcass weight (by 2.02%) with decreased meat lipid content (by 0.33%) in SP group pigs compared to C

BW: body weight, CAZymes: Carbohydrate-Active enZymes, L: lysozyme, SBM: soybean meal, d: day, C: SBM-based diet (Control), TTAD: total tract apparent digestibility, CP: crude protein, CF: crude fat, ADFI: average daily feed intake, ADG: average daily gain, FCR: feed conversion ratio, ADF: acid detergent fiber, SFA: saturated fatty acid, PUFA: polyunsaturated fatty acid, Chol LDL: low-density lipoprotein cholesterol, DM: dry matter, AE: apparent energy, MJ: mega joule.

**Table 3:** Chemical and nutritional composition of black soldier fly (BSF, *Hermetia illucens*) and *Tenebrio molitor* larvae (%) compared to conventional soybean meal (SBM).

	*Hermetia illucens* Larvae			*Tenebrio molitor* Larvae			
Proximate composition	FF	FF	DF	FF	FF	DF	SBM
Moisture	7.78	61.90	6.10	8.3		8.50	11.00
Crude protein	34.97	43.10	40.80	52.4	53.22	68.00	43.80
Crude fat	35.49	38.60	12.80	28.0	34.54	4.00	1.50
Crude ash	6.46	2.70	6.80	8.8	4.04	8.00	
Crude fiber			14.3		6.26		
Neutral detergent fiber (NDF)			13.3				13.30
NDF-Nitrogen			5.8				
Acid detergent fiber (ADF)	6.71		9.1				9.40
ADF-linked protein	5.59						
Starch			14.0		1.94		
Chitin	4.65	6.70					
Calcium	4.39	0.12	0.58		0.08	0.60	0.32
Phosphorus	0.83	0.41	0.76		1.04	0.50	0.65
Potassium		0.59	1.28		0.74		1.96
Sodium		0.07	0.25		0.11		0.01
Magnesium		0.21	0.33		0.32		0.27
Gross energy (MJ/kg)	21.80		17.8	24.4			17.36
Essential amino acids					20.14	37.76	
Methionine	0.50	0.71	0.77	1.01		2.07	0.61
Methionine + Cystine	0.62						
Cystine		0.22	0.39	1.25	3.16	0.87	0.70
Valine	1.86	2.82	1.76	2.82	2.94	4.78	2.06
Isoleucine	1.36	1.91	1.29	2.21	1.98	3.41	1.99
Leucine	2.12	3.06	1.99	3.15	3.37	7.07	3.42
Tryptophan	0.60	0.54	1.05				0.61
Phenylalanine	1.18	1.64	1.15	1.88	1.76	3.74	2.18
Histidine	0.80	1.38	0.79	1.68	2.80	1.49	1.17
Lysine	2.10	2.30	1.63	3.59	2.01	5.82	2.83
Threonine	1.17	1.62	1.05	1.85	1.83	4.10	1.73
Arginine	2.30	1.99	1.37	2.80	2.23	6.01	3.23
Non-essential amino acids					24.36	45.99	
Alanine	2.55	2.78	2.13	3.89	3.96	4.90	
Aspartic acid	2.67	3.69	2.68	4.37	2.76	7.66	
Glutamic acid	4.11	4.58	3.99	6.29	5.78	13.46	
Glycine	1.61	2.52	1.61	2.21	2.61	6.12	
Serine	1.16	1.59	1.31	2.27	2.20	6.18	
Tyrosine	1.63			3.28	3.45	3.41	1.69
Proline		2.51	2.04	3.43	1.66	1.66	
Fatty acids (% fatty acid methyl esters)							
Lauric acid (C12:0)		57.56			0.11		
Myristic acid (C14:0)		7.14			1.63		
Pentadecanoic acid (C15:0)					0.02		
Palmitic acid (C16:0)		1.03			4.71		
Palmitoleic acid (C16:1)		3.34			0.89		
Stearic acid (C18:0)		0.98			0.08		
Oleic acid (C18:1 n-9)		7.97			15.56		
Linoleic acid (C18:2 n-6)		7.83			7.57		0.69
Linolenic acid (C18:3 n-3)		1.10			0.11		
γ-Linoleic acid (C18:3 n-6)					0.01		
Erucic acid (C22:1)					0.01		
Saturated fatty acid (SFA)		78.29			6.94		
Monounsaturated fatty acid (MUFA)		11.99			16.58		
n-6 PUFA		8.00			7.67		
n-3 PUFA		1.43			0.11		
References	[3] ^1^	[16] ^2^	[12] ^1^	[63] ^3^	[71] ^3^	[65] ^1^	[69] ^1^

^1^ % as- fed basis, ^2^ BSF prepupae reared on restaurant waste (%, dry matter basis, DM), ^3^ DM basis, FF: full-fat, DF: defatted.

**Table 4:** Effect of the replacement of soybean meal with either Spirulina or partly defatted *Hermetia illucens* larval meal in barrows’ diets (Pietrain × (Large White × Landrace)) on the meat’s physical and chemical characteristics [76].

Parameter	SBM-Based Diet (C) (n = 16)	*Hermetia illucens* Diet (n = 15)	Spirulina Diet (n = 16)
Carcass weight (kg) ^1^	95.08 (1.17)	97.99 (1.21)	93.11 (1.17)
Lean meat yield (%) ^1^	59.52 (0.45)	58.77 (0.46)	59.05 (0.45)
GM (*M. glutaeus medius*) pH_45min_	6.08 (0.05)	6.24 (0.05)	5.97 (0.05)
LTL (*Longissimus thoracis et lumborum*) pH_45min_	5.91 (0.08)	6.05 (0.09)	6.04 (0.09)
LTL (*Longissimus thoracis et lumborum*) pH_u_	5.41 (0.02)	5.40 (0.03)	5.43 (0.03)
Cooking loss (%)	32.4 (0.30)	31.3 (0.30)	32.4 (0.30)
Instrumental tenderness (N)	10.78 (0.27)	10.49 (0.30)	10.51 (0.29)
Protein (%)	23.10 (0.12)	23.05 (0.13)	22.93 (0.13)
Intramuscular fat (%)	2.96 (0.19)	3.27 (0.21)	3.04 (0.20)
Water content (%)	72.60 (0.16)	72.02 (0.18)	72.44 (0.17)
Backfat L* (lightness)	79.09 ^a^ (0.27)	78.17 ^b^ (0.30)	78.72 ^ab^ (0.29)
Backfat a* (redness)	4.09 (0.18)	4.26 (0.19)	4.02 (0.19)
Backfat b* (yellowness)	4.85 (0.20)	5.15 (0.22)	5.52 (0.21)
Lean L* (lightness)	63.18 (0.89)	61.62 (0.97)	62.96 (0.93)
Lean a* (redness)	2.74 (0.23)	3.56 (0.25)	2.94 (0.24)
Lean b* (yellowness)	13.84 (0.30)	13.77 (0.33)	13.71 (0.32)
TBARS (μg/g)	0.357 (0.043)	0.399 (0.047)	0.470 (0.047) ^2^

^a,b^ Different superscript letters indicate statistical differences between groups at *p* < 0.05; the absence of letters indicates no significant difference. ^1^ Calculated without considering carcass weight as a covariate. * CIELAB L*a*b* scale [6]. ^2^ n = 15.

**Table 5:** Effects of insect larvae meal supplementation in pig diets.

References	Phase	Breed	Sex	Ingredient Type	Supplementation Level (% As-Fed Basis)	Age	Performance
[100]	Fattening pigs	Duroc × Landrace × Large White	Female	FF BSFLM	4 (H1 diet), 8 (H2 diet)	Initial BW 76.0 kg (46-day feeding period)	H1 diet increased ADG and decreased FCR compared with SBM-based and H2 diets. No difference in ADFI H1 and H2 increased *Lactobacillus Pseudobutyrivibrio*, *Roseburia*, *Faecalibacterium*, *Clostridium* cluster XIVa bacteria and decreased the abundance of *Streptococcus* Improved immune status of the intestinal mucosa of pigs, through an alteration of the bacterial composition and its metabolites
[103]	Fattening pigs	Duroc × Landrace × Large White	Female	FF BSFLM	4 (HI4 diet), 8 (HI8 diet)	Initial BW 76.0 kg (46-day feeding period)	HI4 diet increased final BW compared to HI8 and SBM diet (HI0) No difference in the 45 min and 24 h L*, a*, and b* parameters, drip loss, or shear force between groups HI4 and HI8 diets increased loin-eye area, marbling scores, and inosine monophosphate content in *Longissimus thoracis* (LT) muscle compared to HI0 Increased intramuscular fat content in HI4 group compared to HI0 group Increased expression levels of lipogenic genes and MyHC-IIa mRNA with HI4 diet compared to HI0
[76]	Fattening pigs	Pietrain × (Large White × Landrace)	Barrows	BSFLM (partially DF)	8.16, 12.25 (25–50 kg) 6.50, 9.74 (51–75 kg) 9.5 (>75 kg)	25–50 kg, 51–75 kg, >75 kg	No difference in the physico-chemical and sensory quality of pork packaged under current industrial conditions Increased PUFA and C12:0 (five times) contents in the backfat from group fed BSFLM
[74]	Weaned piglets	-	Males and females	FF and DF BSF prepupae	4 (BSF4), 8 (BSF8) 5.4 (DF-BSF)	6.178 kg (15-day experimental period)	No difference in ADG, ADFI, or FCR Log reductions of only 0.5-fold for D-streptococci in the gut of piglets fed BSF diets No difference in ATTD of nutrients Increased CP AID values in piglets fed BSF4 and DF-BSF diets compared to piglets fed SBM diet; decreased values for BSF8 compared to SBM diet
[102]	Fattening pigs	Large White × Landrace	Boars and gilts	FF BSFLM	6 (D25) 9 (D50) 12 (D75) 14 (D100)	54.3 kg (98-day feeding)	Increased ADG in D50, D75, and D100 groups compared to FM group Increased final BW in D50 and D100 groups compared to FM and D25 Decreased FCR in D50, D75 and D100 groups compared to FM and D25 groups Increased carcass weights in D50, D75 and D100 groups compared to FM group High CP contents (on a dry matter basis) in the heart (65%) and in the lung (93%) in all dietary treatments; higher CF contents in tissues of pigs from D50, D75 and D100 groups than from D0 and D25 ones Prospect of a reduction in pig feeding costs
[12]	Growing pigs	Yorkshire × Landrace × Duroc	Barrows	FF and DF BSFLM	50 (FF), 36.5 (DF)	Two experiments Exp. 1: Initial BW 25.1 kg for AA SID determinations Exp. 2: Initial BW 23.4 kg for NE determination	No difference in CP and Lys SID between FF (80.2, 86.8, resp.) and DF BSFLM (81.0, 89.1, resp.) DE (4927 vs. 3941 ± 75 kcal/kg), ME (4569 vs. 3396 ± 102 kcal/kg), NE (3477 vs. 2640 ± 30 kcal/kg; 3479 vs. 2287 ± 28 kcal/kg, calculated by Noblet or Blok equations, resp.) higher for FF than for DF BSFLM Both FF and DF BSFLM would be potential alternative protein sources
[65]	Growing pigs.	(Landrace × Yorkshire) × Duroc)	-	Mealworm (Tenebrio molitor) larvae hydrolysate (HML) Dried defatted mealworm larvae meal (DMLM)	10.0% (HML, DMLM) compared with: 10% fermented poultry by-product (FPBM) 10% hydrolyzed fish soluble (HFS)	28.70 ± 0.32 kg (two-week recovery period)	The highest DM AID in pigs fed HML diet Higher CP AID in pigs fed HML and DMLM diets than in FPBM and HFS-fed pigs Higher AA AIDs in pigs fed HML diet Similar AIDs of Lys, Met, and Thr in pigs fed DMLM and HML diets, but higher than those in pigs fed FPBM or HFS diet Higher SIDs of DM and CP in Pigs fed HML diet than in pigs fed FPBM and HFS diets No difference in SIDs of total AAs between treatments Higher SIDs of Lys, Met, and Thr in pigs fed HML and DMLM diets than FPBM and HFS diets Higher digestibility in DM, CP, Lys, Met, and Thr in HML-fed pigs than FPBM and HFS diets

BSFLM: black soldier fly larvae meal (*Hermetia illucens*), FF: full-fat, DF: defatted, ADFI: average daily feed intake, ADG: average daily gain, FCR: feed conversion ratio, SBM: soybean meal, BW: body weight, DM: dry matter, PUFA: polyunsaturated fatty acid, FM: fishmeal, AA: amino acids, AID: apparent ileal digestibility, ATTD: apparent total tract digestibility, SID: standardized ileal digestibility, CP: crude protein, CF: crude fat, Lys: lysine, Met: methionine, Thr: threonine, DE: digestible energy, ME: metabolizable energy, NE: net energy.

**Table 6:** Chemical and nutritional composition of grain legumes and rapeseed meal compared to conventional soybean meal (SBM) (g/kg dry matter, DM) [4].

Proximate Composition (g/kg DM)	Pea (Tarchalska)	Rapeseed Meal	Yellow Lupin (Mister)	SBM
Crude protein	213	349	433	463
Acid detergent fiber	92	187	191	79
Neutral detergent fiber	153	259	245	112
Crude fat	18	33	32	19
Starch	578	-	-	-
Essential amino acids (g/16 g N)				
Arginine	12.1	6.03	15.2	7.54
Cysteine	0.89	2.61	1.78	1.35
Glycine	4.18	5.48	4.22	4.29
Histidine	2.78	2.75	3.08	2.51
Isoleucine	3.92	3.39	4.38	4.63
Leucine	7.12	7.14	8.81	7.25
Lysine	7.85	5.23	6.68	5.59
Methionine	0.52	2.05	0.51	1.17
Phenylalanine	4.51	4.28	4.47	4.82
Threonine	3.52	4.63	3.66	3.48
Valine	4.52	5.10	4.09	4.15
Alanine	4.12	4.42	3.38	4.28
Aspartic acid	12.3	7.99	11.6	11.9
Glutamic acid	15.9	15.4	25.0	18.5
Proline	3.74	6.28	3.52	5.25
Serine	4.40	4.49	5.56	5.00
Tyrosine	2.63	3.10	3.22	3.40
Antinutrients				
Total alkaloids	-	-	0.0011	-
RFO ^1^	62.85	20.5	134.2	46.62
Raffinose	8.30	3.49	10.2	7.72
Stachyose	27.10	17.1	82.8	35.1
Verbascose	27.45	-	41.2	3.08
Phytic P	1.86	7.75	4.92	4.11
Tannin	0.025	-	-	-

^1^ RFO: raffinose type oligosaccharides.

**Table 7:** Effects of rapeseed meal (RSM) and legume grains in pig diets.

References	Phase	Breed	Sex	Ingredient Type	Inclusion Level (% As-Fed Basis or Otherwise Stated)	Age	Performance
[4]	Growing-finishing pigs	DanBred hybrid	Male and female	P (*Pisum sativum*, Tarchalska variety) Yellow L (*Lupinus luteus*, Mister variety) RSM	Two feeding periods: Growing: 6 (P) 6 (L) Finishing: 5 (RSM) 5 (P) 4 (L)	30 kg (83 d growing-finishing period)	No interaction between sex and diet Decreased ADG and final BW in pigs fed diets with total SBM replacement No difference in pork meat quality Reduced fatness in pigs fed P, L, and RSM diets compared to C Total SBM dietary replacement decreased growth performance without affecting meat quality
[5]	Growing-finishing pigs	(Landrace × Yorkshire) × Duroc	Male and female	RSM (Mestilla) FB (Columbo)	C: 0.0 (FB), 6 (RSM) RSM/FB: 18 (RSM) 16.112 (FB)	27.7 ± 2.9 (108.7 ± 4.2 kg final BW)	Increased FCR in the finishing period Lower lightness and yellowness meat values No difference in total SFA, MUFA, or PUFA in pigs fed RSM/FB diet RSM/FB diet reduced levels of β-alanine, glucose, and oxidized metabolites (glycine and pyroglutamic acid) in pre-rigor muscle (less oxidative stress, improved cell’s defense mechanisms) Desirable sensory characteristics of pork from pigs fed RSM/FB diet RSM/FB did not compromise growth performance or meat quality
[24]	Growing-finishing pigs	Camborough	Barrows (castrates) and gilts	CM Chickpea (Kabuli and Desi type) P	Three experimental diets in two periods, growing (G) and finishing (F) G: CM (11.65, 11.75, 6.8); Ch and P (30) in the three experimental diets F: CM (4.35, 5.40, 2.60); Ch and P (30) in the three experimental diets	19.91 ± 2.30 kg Growing period: 19.9 ± 61.7 kg Finishing period: 61.7 ± 92.5 kg	Decreased DM and E CUDs in Desi- compared to Kabuli-supplemented pigs No differences in DM and E CUDs between Kabuli, C, and P diets Lower CP CUD in Chick and P diet than in C Decrease ADG in Desi and Kabuli diets compared to C during the growing period No effect of dietary treatment on pig performance during the finishing period and over the whole experimental period No differences in carcass characteristics between experimental groups No negative effects of Desi or Kabuli chickpeas on pig ADG up to 30% inclusion level
[28]	Growing pigs	Duroc × Landrace × Yorkshire	-	Miscellaneous meals (RSM, cottonseed meal, sunflower seed meal)	Two experimental diets CSM (corn–SBM–miscellaneous meal) and CM (corn–miscellaneous meal) 4.04 (in 1:1:1 ratio between meals, partially replacing 10.99% SBM in C diet, on a dry matter basis) 7.69 (in 1:1:1 ratio between meals, completely replacing SBM in C diet, on a dry matter basis)	50.64 ± 2.09 kg (75 kg final BW, 24 d experimental period)	No difference in ADG, ADFI, or FCR No difference in CP, CF, or GE CUDs Increased serum values of ALT and TG, but reduced PUN in CM group compared to C one No effects on serum free AAs CM diet decreased Euryachaeota abundance in fecal microbiota compared to C RSM, cottonseed meal, and sunflower seed meal as potential alternative feed ingredients to SBM in swine diets
[29]	Growing-finishing pigs	Norwegian Landrace pigs	Males and females	RSM	20	24.9 ± 1.98 kg (109.7 kg ± 5.44 final BW, 88 d experimental period)	Reduced ADG and dressing percentage in pigs fed RSM compared to SBM-fed ones No difference in meat quality Changes in gene expression of skeletal muscle from pigs fed RSM diet Negative action of growth regulators in RSM pigs Increased expression of different muscle genes implicated in glucose and lipid metabolism, and mitochondrial function in pigs fed RSM More pronounced proteolysis and reduced protein synthesis in muscle tissue of RSM pigs Increased expression of genes regulating lipolysis, FA oxidation, and oxidative stress in muscle of pigs fed RSM Deepening of knowledge of the molecular mechanisms underlying phenotypic observations
[30]	Growing pigs	-	-	RSM	20	17.8 ± 2.7 kg (3-week experimental period)	Sinapine, sinapic acid, and gluconapin digesta exposure markers of RSM diet Reduced AA AID in RSM-fed pigs No difference in the digesta microbial metabolites (short-chain FA and secondary bile acids) Increased levels of multiple oxidized metabolites and aldehydes while decreased levels of ascorbic acid and docosahexaenoic acid-containing lipids in the liver and serum (possible alteration of the redox balance in RSM-fed pigs) Need of processing treatments to improve RSM use in pig diets
[111]	Growing pigs	-	-	Opal Chick meal Dehulled Tyson Chick meal Dehulled P meal	250, 500, 750 g/kg	20–50 kg (experimental period)	No difference in ADG between pigs fed the two Chick meals or C diet Dehulled P meal linearly decreased ADG and ADFI and increased the FCR No effect of Chick meals on organ weights; effect of P meal on liver and pancreas weights (presence of other anti-nutritional factors) Dietary tolerance levels of at least 4.7 and 4.5 mg/g of TI and CI, respectively, in growing pigs Threshold levels unlikely to be exceeded in conventional diets based on most grain legumes
[112]	Piglets	-	-	FB with different content of condensed tannins (T): white flowering variety (low-TFB, <0.1%) (cv ‘Blandine’); colored flowering variety (medium-TFB, 0.4%) (cv ‘Herz Freya’); high tannin content (high-TFB, 1.0%) (cv ‘Mythos’ and ‘Alfred’) low-TFB: raw or autoclaved (30 min, 105 °C).	30	15–30 kg	Increased CP and most AAs’ AID for the low-TFB (white flowering variety) compared to the medium- and high-TFB Difference in CP and AA ATTD between ‘Blandine’ and ‘Alfred’ cv Autoclaving the low-TFB ‘Blandine’ did not improve CP and AA AID and TTAD No difference in starch AID for all raw and autoclaved beans. Autoclaving low-TFB did not improve their nutritional value for pigs
[113]	Growing-finishing pigs	Polish Large White × Duroc	Barrows	FB (cv. Caspar, Nadwislahski) with various levels of condensed tannins (T), high (HT), low (LT), dehulled (DHT) and added white flowering-P hull fiber (DHTF)	30	25–63 kg BW (54 d experiment)	Increased N-free extractives and E CUD in the LT and DHT diets compared to the HT diet No difference between groups in daily nitrogen retention and utilization No difference between groups in ADG and FCR HT diet (1 g/kg phenols and 0.6g/kg proanthocyanidins) did not decrease ADG of 25–63 kg pigs (slightly under 0.700 kg/d) compared to LT and DHT diets.
[114]	Pigs	-	Male	Expt. 1: *L. angustifolius* (seeds or kernels) and P Expt. 2, 3: *L. angustifolius* (seeds or kernels); *L. albus* (seeds or kernels), P	Expt. 1: 40 Expt. 2, 3: 35	Expt. 1: 45 kg (14 d expt. period) Expt. 2: 60 kg (14 d expt. period) Expt. 3: 75 kg (28 d expt. period)	Increased retention times in *L. angustifolius* diets (both seeds or kernels) compared to wheat-alone diet; intermediate value in P diet Decreased transit time of high levels lupin-contained diets, more for *L. albus*, with predictable reduced ADFI and ADG Suggested physical treatment or enzymatic supplementation for *L. albus* diets to increase ADFI as well as appropriate genetic selection against specific NSPs
[115]	Growing pigs	-	-	Field peas not heat-treated (C) or extruded at 75, 115, or 155 °C or pelleted at 75 °C.	40	69.3 ± 2.9 kg (9-d expt. period)	Extrusion of P increases the AID of CP, AA, starch, and E and E ATTD No effects of pelleting P on nutrient and E AIDs but only on the E ATTD.
[116]	Early-weaned pigs	Cotswold	-	P (raw, extruded and micronized) with or without enzyme supplementation (amylase and xylanase)	40	4.5 ± 0.5 kg (16-d)	Extrusion and micronization of amylase and xylanase supplemented P-based diets improved AA AIDs compared to raw P Micronization improved lysine AID (82 vs. 91%) No difference in ADFI and ADG Increased FCR (1.4 vs. 1.20) in phase 1 pigs (4.5–10.0 kg BW) fed raw P-based diet supplemented with amylase and xylanase Decreased PUN levels in pigs fed raw P-based diet supplemented with enzymes
[117]	Growing-finishing pigs	-	-	P in an expanded-processed P-based diet or as the only expanded or extruded component of a P-based diet Expanded diets: 105 °C for 5 s at 35 bar pressure P: extruded at 130 °C for 30 s and expanded at 130 °C for 10 s at 42 bar pressure	40		Reduced OM, CP, and E CUDs in pigs fed P-based diet compared to C Expansion did not affect nutrient CUDs Reduced ADG and increased FCR (34 kg to slaughter) in pigs fed P-based diets compared to C diets Expansion did not influence ADG or FCR OM, CP, and E CUDs differed due to dietary treatments (C, raw, expanded, or extruded P) ADG and FCR also differed due to dietary treatments (C, raw, expanded, or extruded P) No effect of expansion on feed nutritive value
[118]	Piglets	Expt. 1: Polish Landrace Expt. 2: Dutch Landrace × Dutch Yorkshire	castrated male pigs	Yellow lupin (*Lupinus luteus* L.) cvs Juno and Amulet (*Lupinus angustifolius*) cv Saturn with or without exogenous α-galactosidase (α-G) (5 g/kg)	30.7 (*L. luteus* cv Juno, DM basis) 35.0 (*L. luteus* cv Amulet, DM basis) 35.0 (*L. angustifolius* cv Saturn, DM basis)	Exp. 1: 18–24 kg Exp. 2: 11–15 kg	Exp. 1: increased CUD of galactosides in cv Juno with α-galactosidase supplementation (97 vs. 80%) Exp. 2: increased AID of sucrose (92.9%), stachyose (7 1.9%) and verbascose (83.4%) in *L. angustifolius* (cv Saturn) compared to yellow L (cv Amulet) (86.3, 60.1, and 77.8%, respectively) AID of raffinose 52.2% for cv Saturn and 40.7% for cv Amulet. AID of total NSP by 11% for cv Amulet and by 14% for cv Saturn. α-G supplementation improved CUDs of the raffinose series oligosaccharides with positive effects on the AID of most AAs
[119]	Growing-finishing pigs.	(Landrace × Large White)	-	Extruded Chick (ECKP) (120 °C for 20 s, variety ‘Serifos’)	10 (8 SBM, ECKP1) 20 (0 SBM, ECKP2) 30 (0 SBM, ECKP3)	53 ± 4 d (17-week study) (53–94 d growing period) (95–171 d finishing period)	Small differences between C and experimental diets in chemical composition No difference between groups in meat FA composition, pH, and color Lower cooking loss in the C group than the others Slightly greater scores for tenderness and juiciness for C group compared to Chick treatments No differences in taste scores Replacement of SBM with ECKP in isonitrogenous and isoenergetic diets at inclusion levels up to 30% does not affect meat quality
[120]	Growing-finishing pigs.	Landrace, Landrace × Yorkshire	Gilts and barrows	RSM, FB (cv. Kontu)	R diet: 22 (RSM), 0.0 (FB) F 25 diet: 16.5 (RSM), 13.7 (FB); F 50 diet: 11.0 (RSM), 19.7 (FB); F 75 diet: 11.0 (RSM), 19.7 (FB); F 100 diet: 0.0 (RSM), 31.7 (FB); 24.3 (FB) (0.0 RSM Expt. 2)	25–110 kg	Quadratic effect on ADG and on FCR by replacing RSM with FB in the growing and during total fattening period Decreased ADG of growing pigs when FB replaced 75 or 100% of RSM Decreased ADFI by complete replacing RSM with FB Best overall ADG and FCR by replacing 50% RSM with FB Darker meat with increasing dietary FB inclusion levels Higher ultimate pH, but no difference in meat color, of the *Longissimus dorsi* muscle of pigs from FB diet (24.3%) compared to C (without RSM) Reduced ADG of growing pigs by inclusion level of FB over 200 g/kg in a barley + RSM based diet
[121]	Growing-finishing pigs	(Large white × Landrace) × duroc	Male and female	RSM (Greek origin)	16.7, 10.6 (growing and fattening period, respectively)	3 months (90 d expt. period) Growing (1–30 d) Fattening (31–90 d)	No difference in ADG during the growing and fattening periods No difference in FCR between groups during the growing period but greater value for RSM-fed pigs than C-fed pigs during the fattening period Some differences in meat chemical composition (M, CP, CF) between groups Greater total MUFA and lower SFA and PUFA contents in the steak cut from RSM group than C No differences on the ham and steak meat lipid oxidative stability after 4 or 7 d of refrigeration (4 °C) Greater *Lactobacillus* spp. in the caecum and lower *Clostridium perfringens* in the mid-colon from RSM group than C RSM of Greek origin as a viable, cheaper, and eco-friendly alternative to imported SBM in pig diets

ADFI: average daily feed intake, ADG: average daily gain, FCR: feed conversion ratio, P: pea, L: lupin, FB: fava beans, CM: canola meal, Chick: chickpea, TI: trypsin inhibitor, CI: chymotrypsin inhibitor, DM: dry matter, GE: gross energy, E: energy, NSP: non-starch polysaccharide, CUD: digestibility utilization coefficient, AID: apparent ileal digestibility, ATTD: apparent total tract digestibility, AA: amino acid, CP: crude protein, CF: crude fat, OM: organic matter, M: moisture, SBM: soybean meal, C: SBM-based diet (Control), PUN: plasma urea nitrogen, ALT: alanine aminotransferase, TG: triglyceride, FA: fatty acid, SFA: saturated fatty acid, MUFA: monounsaturated fatty acid, PUFA: polyunsaturated fatty acid, d: day, BW: body weight.

## Data Availability

Not applicable.

## References

[1] Food and Agriculture Organization of the United Nations (FAO) (2011). World Livestock 2011—Livestock in Food Security.

[2] Hong J., Han T., Yong Kim Y. (2020). Mealworm (*Tenebrio molitor* Larvae) as an Alternative Protein Source for Monogastric Animal: A Review. Animals.

[3] Lu S., Taethaisong N., Meethip W., Surakhunthod J., Sinpru B., Sroichak T., Archa P., Thongpea S., Paengkoum S., Aprilia R. (2022). Nutritional Composition of Black Soldier Fly Larvae (*Hermetia illucens* L.) and its Potential Uses as Alternative Protein Sources in Animal Diets: A Review. Insects.

[4] Zmudzińska A., Bigorowski B., Banaszak M., Roślewska A., Adamski M., Hejdysz M. (2020). The Effect of Diet Based on Legume Seeds and Rapeseed Meal on Pig Performance and Meat Quality. Animals.

[5] Grabež V., Egelandsdal B., Kjos N.P., Håkenåsen I.M., Mydland L.T., Vik J.O., Hallenstvedt E., Devle H., Øverland M. (2020). Replacing soybean meal with rapeseed meal and faba beans in a growingfinishing pig diet: Effect on growth performance, meat quality and metabolite changes. Meat Sci..

[6] Lestingi A. (2023). Use of Wild Boar (*Sus scrofa*) as a Sustainable Alternative in Pork Production: A Review. Animals.

[7] Holman B.W.B., Malau-Aduli A.E.O. (2013). Spirulina as a livestock supplement and animal feed. J. Anim. Physiol. Anim. Nutr..

[8] Poppi D.P., McLennan S.R. (2010). Nutritional research to meet future challenges. Anim. Prod. Sci..

[9] Martins C.F., Pestana Assunção J., Ribeiro Santos D.M., Madeira M.S.M.d.S., Alfaia C.M.R.P.M., Lopes P.A.A.B., Coelho D.F.M., Cardoso Lemos J.P., de Almeida A.M., Mestre Prates J.A. (2021). Effect of dietary inclusion of Spirulina on production performance, nutrient digestibility and meat quality traits in post-weaning piglets. J. Anim. Physiol. Anim. Nutr..

[10] Manceron S., Ben-Ari T., Dumas P. (2014). Feeding proteins to livestock: Global land use and food vs. feed competition. OCL Oilseeds Crops Fats Lipids.

[11] Al-Yahyaey F., Al-Marzooqi W., Shaat I., Smith M.A., Al-Sabahi J., Melak S., Russell D.B. (2023). Effect of *Spirulina platensis* Supplementation on Carcass Characteristics, Fatty Acid Profile, and Meat Quality of Omani Goats. Animals.

[12] Crosbie M., Zhu C., Shoveller A.K., Huber L.-A. (2020). Standardized ileal digestible amino acids and net energy contents in full fat and defatted black soldier fly larvae meals (*Hermetia illucens*) fed to growing pigs. Transl. Anim. Sci..

[13] Wang Y.-S., Shelomi M. (2017). Review of black soldier fly (*Hermetia illucens*) as animal feed and human food. Foods.

[14] Makkar H.P., Tran G., Heuzé V., Ankers P. (2014). State-of-the-art on use of insects as animal feed. Anim. Feed Sci. Technol..

[15] Nowak V., Persijn D., Rittenschober D., Charrondiere U.R. (2016). Review of food composition data for edible insects. Food Chem..

[16] Spranghers T., Ottoboni M., Klootwijk C., Ovyn A., Deboosere S., De Meulenaer B., Michiels J., Eeckhout M., De Clercq P., De Smet S. (2016). Nutritional composition of black soldier fly (*Hermetia illucens*) prepupae reared on different organic waste substrates. J. Sci. Food Agric..

[17] Nedeva R., Jordanova G., Kistanova E., Shumkov K., Georgiev B., Abadgieva D., Kacheva D., Shimkus A., Shimkine A. (2014). Effect of the addition of *Spirulina platensis* on the productivity and some blood parameters on growing pigs. Bulg. J. Agric. Sci..

[18] Aly M.S., Amber S.G., El-Sayed M.K. (2011). Production and application of *Spirulina platensis* rich in fatty acids, and vitamins. J. Am. Sci..

[19] Grinstead G.S., Tokach M.D., Dritz S.S., Goodband R.D., Nelssen J.L. (2000). Effects of *Spirulina platensis* on growth performance of weanling pigs. Anim. Feed Sci. Technol..

[20] Belay A., Yoshimichi O., Miyakawa K., Shimamatsu H. (1993). Current knowledge on potential health benefits of Spirulina. J. Appl. Phycol..

[21] Spolaore P., Joannis-Cassan C., Duran E., Isambert A. (2006). Commercial applications of microalgae. J. Biosci. Bioeng..

[22] Lum K., Kim J., Lei X. (2013). Dual potential of microalgae as a sustainable biofuel feedstock and animal feed. J. Anim. Sci. Biotechnol..

[23] Peiretti P., Meineri G. (2011). Effects of Diets with Increasing Levels of Spirulina Platensis on the Carcass Characteristics, Meat Quality and Fatty Acid Composition of Growing Rabbits. Livest. Sci..

[24] Mustafa A.F., Thacker P.A., McKinnon J.J., Christensen D.A., Racz V.J. (2000). Nutritional value of feed grade chickpeas for ruminants and pigs. J. Sci. Food Agric..

[25] Lestingi A., Facciolongo A.M., Jambrenghi A.C., Ragni M., Toteda F. (2016). The use of peas and sweet lupin seeds alone or in association for fattening lambs: Effects on performance, blood parameters and meat quality. Small Rumin. Res..

[26] Lestingi A., Facciolongo A.M., De Marzo D., Nicastro F., Toteda F. (2015). The use of faba bean and sweet lupin seeds in fattening lamb feed. 2. Effects on meat quality and fatty acidc omposition. Small Rumin. Res..

[27] Lestingi A., Toteda F., Vicenti A., De Marzo D., Facciolongo A.M. (2015). The Use of Faba Bean and Sweet Lupin Seeds Alone or in Combination for Growing Lambs. 1. Effects on Growth Performance, Carcass Traits, and Blood Parameters. Pak. J. Zool..

[28] He Z., Zhan X., Cao S., Wen X., Hou L., Liu S., Zheng H., Gao K., Yang X., Jiang Z. (2023). Effect of Miscellaneous Meal Replacements for Soybean Meal on Growth Performance, Serum Biochemical Parameters, and Gut Microbiota of 50–75 kg Growing Pigs. Animals.

[29] Skugor A., Kjos N.P., Sundaram A.Y.M., Mydland L.T., Ånestad R., Anne-Helene Tauson A.-H., Øverland M. (2019). Effects of long-term feeding of rapeseed meal on skeletal muscle transcriptome, production efficiency and meat quality traits in Norwegian Landrace growing-finishing pigs. PLoS ONE.

[30] Chen C., Pérez de Nanclares M., Kurtz J.F., Trudeau M.P., Wang L., Yao D., Saqui-Salces M., Urriola P.E., Mydland L.T., Shurson G.C. (2018). Identification of redox imbalance as a prominent metabolic response elicited by rapeseed feeding in swine metabolome. J. Anim. Sci..

[31] Jezierny D., Mosenthin R., Bauer E. (2010). The use of grain legumes as a protein source in pig nutrition: A review. Anim.Feed Sci. Technol..

[32] Vadivel V., Pugalenthi M. (2008). Effect of various processing methods on the levels of antinutritional constituents and protein digestibility of *Mucuna pruriens* (L.) DC. var. utilis (Wall. ex Wight) Baker ex Burck (velvet bean) seeds. J. Food Biochem..

[33] Hanczakowska E., Swiatkiewicz M. (2013). Legume seeds and rapeseed press cake as substitutes for soybean meal in sow and piglet feed. Agric. Food Sci..

[34] Hanczakowska E., Świątkiewicz M. (2014). Legume seeds and rapeseed press cake as replacers of soybean meal in feed for fattening pigs. Ann. Anim. Sci..

[35] Ravindran R., Koopmans S., Sanders J.P.M., McMahon H., Gaffey J. (2021). Production of Green Biorefinery Protein Concentrate Derived from Perennial Ryegrass as an Alternative Feed for Pigs. Clean Technol..

[36] Kitada K., Machmudah S., Sasaki M., Goto M., Nakashima Y., Kumamoto S., Hasegawa T. (2009). Antioxidant and antibacterial activity of nutraceutical compounds from Chlorella vulgaris extracted in hydrothermal condition. Sep. Sci. Technol..

[37] Hoseini S.M., Khosravi-Darani K., Mozafari M.R. (2013). Nutritional and medical applications of Spirulina Microalgae. Mini-Rev. Med. Chem..

[38] Lugarà R., Realini L., Kreuzer M., Giller K. (2022). Effects of maternal high-energy diet and spirulina supplementation in pregnant and lactating sows on performance, quality of carcass and meat, and its fatty acid profile in male and female offspring. Meat Sci..

[39] Brown M.R., Jeffrey S.W., Volkman J.K., Dunstan G.A. (1997). Nutritional properties of microalgae for mariculture. Aquaculture.

[40] Navarro N., Yúfera M., García-Gallego M. (2001). Use of freeze-dried microalgae for rearing gilthead seabream, *Sparus aurata* L., larvae. II. Biochemical composition. Hydrobiologica.

[41] Martínez-Fernández E., Acosta-Salmón H., Southgate P.C. (2006). The nutritional value of seven species of tropical microalgae for blacklip pearl oyster (*Pinctada margaritifera*, L.) larvae. Aquaculture.

[42] Martínez-Fernández E., Southgate P.C. (2007). Use of tropical microalgae as food for larvae of the black-lip pearl oyster *Pinctada margaritifera*. Aquaculture.

[43] Gutiérrez-Salmeán G., Fabila-Castillo L., Chamorro-Cevallos G. (2015). Nutritional and toxicological aspects of Spirulina (Arthrospira). Nutr. Hosp..

[44] Madeira M.S., Cardoso C., Lopes P.A., Coelho D., Afonso C., Bandarra N.M., Prates J.A.M. (2017). Microalgae as feed ingredients for livestock production and meat quality: A review. Livest. Sci..

[45] Dismukes G.C., Carrieri D., Bennette N., Ananyev G.M., Posewitz M.C. (2008). Aquatic phototrophs: Efficient alternatives to land-based crops for biofuels. Curr. Opin. Biotechnol..

[46] Kulpys J., Paulauskas E., Pilipavicius V., Stankevicius R. (2009). Influence of cyanobacteria Arthrospira (Spirulina) platensis biomass additive towards the body condition of lactation cows and biochemical milk indexes. Agron. Res..

[47] Volkmann H., Imianovsky U., Oliveira J.L.B., Sant’Anna E.S. (2008). Cultivation of Arthrospira (Spirulina) platensis in desalinator wastewater and salinated synthetic medium: Protein content and amino-acid profile. Braz. J. Microbiol..

[48] Chaiklahan R., Chirasuwan N., Siangdung W., Paithoonrangsarid K., Bunnag B. (2010). Cultivation of *Spirulina platensis* using pig wastewater in a semi-continuous process. J. Microbiol. Biotechnol..

[49] Mitchell S.A., Richmond A. (1988). Optimization of a growth medium for Spirulina based on cattle waste. Biol. Waste.

[50] Hasdai A., Ben Ghedalia D. (1981). Sewage-grown algae as a source of supplementary nitrogen for ruminants. J. Agric. Sci..

[51] Gerken H.G., Donohoe B., Knoshaug E.P. (2012). Enzymatic cell wall degradation of Chlorella vulgaris and other microalgae for biofuels production. Planta.

[52] Popper Z.A., Tuohy M.G. (2010). Beyond the green: Understanding the evolutionary puzzle of plant and algal cell walls. Plant Physiol..

[53] Al-Zuhair S., Ashraf S., Hisaindee S., Darmaki N.A., Battah S., Svistunenko D., Reeder B., Stanway G., Chaudhary A. (2016). Enzymatic pre-treatment of microalgae cells for enhanced extraction of proteins. Eng. Life Sci..

[54] Furbeyre H., Milgen J., Mene T., Gloaguen M., Labussière E. (2017). Effects of dietary supplementation with freshwater microalgae on growth performance, nutrient digestibility and gut health in weaned piglets. Animal.

[55] Lallès J.-P., Boudry G., Favier C., Le Floc’h N., Luron I., Montagne L., Oswald I.P., Pié S., Piel C., Sève B. (2004). Gut function and dysfunction in young pigs: Physiology. Anim. Res..

[56] Zhu L., Zhao K., Chen X., Xu J. (2012). Impact of weaning and an antioxidant blend on intestinal barrier function and antioxidant status in pigs. J. Anim. Sci..

[57] Saeid A., Chojnacka K., Korczynski M., Korniewicz D., Dobrzanski Z. (2013). Effect on supplementation of Spirulina maxima enriched with cu on production performance, metabolical and physiological parameters in fattening pigs. J. Appl. Phycol..

[58] Simkus A., Simkiene A., Cernauskiene J., Kvietkute N., Cernauskas A., Paleckaitis M., Kerziene S. (2013). The effect of blue algae *Spirulina platensis* on pig growth performance and carcass and meat quality. Vet. Zootech..

[59] Evans A.M., Smith D.L., Moritz J.S. (2015). Effects of algae incorporation into broiler starter diet formulations on nutrient digestibility and 3 to 21 d bird performance. J. Appl. Poult. Res..

[60] Granaci V. (2007). Achievements in the artificial insemination of swine. Bull. Univ. Agric. Sci. Vet. Med. Cluj-Napoca Anim. Sci. Biotechnol..

[61] Oonincx D.G., De Boer I.J. (2012). Environmental impact of the production of mealworms as a protein source for humans—A life cycle assessment. PLoS ONE.

[62] Ramos-Elorduy J., González E.A., Hernández A.R., Pino J.M. (2002). Use of *Tenebrio molitor* (Coleoptera: Tenebrionidae) to recycle organic wastes and as feed for broiler chickens. J. Econ. Entomol..

[63] De Marco M., Martínez S., Hernandez F., Madrid J., Gai F., Rotolo L., Belforti M., Bergero D., Katz H., Dabbou S. (2015). Nutritional value of two insect larval meals (*Tenebrio molitor* and *Hermetia illucens*) for broiler chickens: Apparent nutrient digestibility, apparent ileal amino acid digestibility and apparent metabolizable energy. Anim. Feed Sci. Technol..

[64] Yoo J.S., Cho K.H., Hong J.S., Jang H.S., Chung Y.H., Kwon G.T., Shin D.G., Kim Y.Y. (2019). Nutrient ileal digestibility evaluation of dried mealworm (*Tenebrio molitor*) larvae compared to three animal protein by-products in growing pigs. Asian-Australas. J. Anim. Sci..

[65] Cho K.H., Kang S.W., Yoo J.S., Song D.K., Chung Y.H., Kwon G.T., Kim Y.Y. (2019). Effects of mealworm (*Tenebrio molitor*) larvae hydrolysate on nutrient ileal digestibility in growing pigs compared to those of defatted mealworm larvae meal, fermented poultry by-product, and hydrolyzed fish soluble. Asian-Australas. J. Anim. Sci..

[66] Jin X.H., Heo P.S., Hong J.S., Kim N.J., Kim Y.Y. (2016). Supplementation of dried mealworm (*Tenebrio molitor* larva) on growth performance, nutrient digestibility and blood profiles in weaning pigs. Asian -Australas. J. Anim. Sci..

[67] Benzertiha A., Kierończyk B., Kołodziejski P., Pruszyńska–Oszmałek E., Rawski M., Józefiak D., Józefiak A. (2020). *Tenebrio molitor* and Zophobas morio full-fat meals as functional feed additives affect broiler chickens’ growth performance and immune system traits. Poult. Sci..

[68] Heidari-Parsa S. (2018). Determination of yellow mealworm (*Tenebrio molitor*) nutritional value as an animal and human food supplementation. Arthropods.

[69] National Research Council (2012). Nutrient Requirements of Swine.

[70] Hussain I., Khan S., Sultan A., Chand N., Khan R., Alam W., Ahmad N. (2017). Meal worm (*Tenebrio molitor*) as potential alternative source of protein supplementation in broiler. Int. J. Biosci..

[71] Ghosh S., Lee S.M., Jung C., Meyer-Rochow V.B. (2017). Nutritional composition of five commercial edible insects in South Korea. J. Asia-Pac. Entomol..

[72] Wu R.A., Ding Q., Yin L., Chi X., Sun N., He R., Luo L., Ma H., Li Z. (2020). Comparison of the nutritional value of mysore thorn borer (*Anoplophora chinensis*) and mealworm larva (*Tenebrio molitor*): Amino acid, fatty acid, and element profiles. Food Chem..

[73] Yi H.Y., Chowdhury M., Huang Y.D., Yu X.Q. (2014). Insect antimicrobial peptides and their applications. Appl. Microbiol. Biotechnol..

[74] Spranghers T., Michiels J., Vrancx J., Ovyn A., Eeckhoutc M., De Clercq P., De Smet S. (2018). Gut antimicrobial effects and nutritional value of black soldier fly (*Hermetia illucens* L.) prepupae for weaned piglets. Anim. Feed Sci. Technol..

[75] Stein H.H., Adeola O., Cromwell G.L., Kim S.W., Mahan D.C., Miller P.S. (2011). Concentration of dietary calcium supplied by calcium carbonate does not affect the apparent total tract digestibility of calcium, but decreases digestibility of phosphorus by growing pigs. J. Anim. Sci..

[76] Altmann B.A., Neumann C., Rothstein S., Liebert F., Mörlein D. (2019). Do dietary soy alternatives lead to pork quality improvements or drawbacks? A look into micro-alga and insect protein in swine diets. Meat Sci..

[77] Jonas-Levi A., Martinez J.J.I. (2017). The high level of protein content reported in insects for food and feed is overestimated. J. Food Compos. Anal..

[78] Huang S.X., Sauer W.C., Marty B. (2001). Ileal digestibilities of neutral detergent fiber, crude protein, and amino acids associated with neutral detergent fiber in wheat shorts for growing pigs. J. Anim. Sci..

[79] Janssen R.H., Vincken J.P., van den Brock L.A.M., Fogliano V., Lakemond C.M.M. (2017). Nitrogen-to-protein conversion factors for three edible insects: *Tenebrio molitor*, Alphitobius diaperinus, and *Hermetia illucens*. J. Agric. Food Chem..

[80] Nery J., Gasco L., Dabbou S., Schiavone A. (2018). Protein composition and digestibility of black soldier fly larvae in broiler chickens revisited according to the recent nitrogen-protein conversion ratio. J. Insects Food Feed.

[81] Gravel A., Doyen A. (2020). The use of edible insect proteins in food: Challenges and issues related to their functional properties. Innov. Food Sci. Emerg. Technol..

[82] Schiavone A., De Marco M., Martínez S., Dabbou S., Renna M., Madrid J., Hernandez F., Rotolo L., Costa P., Gai F. (2017). Nutritional value of a partially defatted and a highly defatted black soldier fly larvae (*Hermetia illucens* L.) meal for broiler chickens: Apparent nutrient digestibility, apparent metabolizable energy and apparent ileal amino acid digestibility. J. Anim. Sci. Biotechnol..

[83] Huang C., Feng W., Xiong J., Wang T., Wang W., Wang C., Yang F. (2018). Impact of drying method on the nutritional value of the edible insect protein from black soldier fly (*Hermetia illucens* L.) larvae: Amino acid composition, nutritional value evaluation, in vitro digestibility, and thermal properties. Eur. Food Res. Technol..

[84] Lee C.G., Da Silva C.A., Lee J.Y., Hartl D., Elias J.A. (2008). Chitin regulation of immune responses: An old molecule with new roles. Curr. Opin. Immunol..

[85] Sánchez-Muros M.J., Barroso F.G., Manzano-Agugliaro F. (2014). Insect meal as renewable source of food for animal feeding: A review. J. Clean. Prod..

[86] Kramer K.J., Hopkins T.L., Schaefer J. (1995). Applications of solids NMR to the analysis of insect sclerotized structures. Insect Biochem. Mol. Biol..

[87] Xu Y.Q., Wang Z.Q., Wang Y.L., Yan S.M., Shi B.L. (2018). Effects of chitosan as growth promoter on diarrhea, nutrient apparent digestibility, fecal microbiota and immune response in weaned piglets. J. Appl. Anim. Res..

[88] Valdés F., Villanueva V., Durán E., Campos F., Avendaño C., Sánchez M., Domingoz-Araujo C., Valenzuela C. (2022). Insects as Feed for Companion and Exotic Pets: A Current Trend. Animals.

[89] Zuk-Gołaszewska K., Gałęcki R., Obremski K., Smetana S., Figiel S., Gołaszewski J. (2022). Edible Insect Farming in the Context of the EU Regulations and Marketing—An Overview. Insects.

[90] Van Huis A. (2020). Insects as food and feed, a new emerging agricultural sector: A review. J. Insects Food Feed.

[91] Attygalle A.B., Blankespoor C.L., Meinwald J., Eisner T. (1991). Defensive secretion of *Tenebrio molitor* (Coleoptera: Tenebrionidae). J. Chem. Ecol..

[92] Vandeweyer D., Milanović V., Garofalo C., Osimani A., Clementi F., Van Campenhout L., Aquilanti L. (2019). Real-time PCR detection and quantification of selected transferable antibiotic resistance genes in fresh edible insects from Belgium and the Netherlands. Int. J. Food Microbiol..

[93] Ravzanaadii N., Kim S.H., Choi W.H., Hong S.J., Kim N.J. (2012). Nutritional value of mealworm, *Tenebrio molitor* as food source. Int. J. Ind. Entomol..

[94] Van Broekhoven S., Gutierrez J.M., De Rijk T.C., De Nijs W.C., Van Loon J.J. (2017). Degradation and excretion of the Fusarium toxin deoxynivalenol by an edible insect, the Yellow mealworm (*Tenebrio molitor* L.). World Mycotoxin J..

[95] Camenzuli L., Van Dam R., De Rijk T., Andriessen R., Van Schelt J., der Fels-Klerx V. (2018). Tolerance and excretion of the mycotoxins aflatoxin B1, zearalenone, deoxynivalenol, and ochratoxin A by Alphitobius diaperinus and *Hermetia illucens* from contaminated substrates. Toxins.

[96] AAFCO (2016). Association of American feed control officials. Proceedings of the AAFCO Annual Meeting Agenda and Committee Reports.

[97] Cutrignelli M.I., Messina M., Tulli F., Randazzo B., Olivotto I., Gasco L., Loponte R., Bovera F. (2018). Evaluation of an insect meal of the black soldier Fly (*Hermetia illucens*) as soybean substitute: Intestinal morphometry, enzymatic and microbial activity in laying hens. Res. Vet. Sci..

[98] Maurer V., Holinger M., Amsler Z., Früh B., Wohlfahrt J., Stamer A., Leiber F. (2016). Replacement of soybean cake by *Hermetia illucens* meal in diets for layers. J. Insects Food Feed.

[99] Borrelli L., Coretti L., Dipineto L., Bovera F., Menna F., Chiariotti L., Nizza A., Lembo F., Fioretti A. (2017). Insect based diet, a promising nutritional source, modulates gut microbiota composition and SCFAs production in laying hens. Sci. Rep..

[100] Yu M., Li Z., Chen W., Rong T., Wang G., Ma X. (2019). *Hermetia illucens* larvae as a potential dietary protein source altered the microbiota and modulated mucosal immune status in the colon of finishing pigs. J. Anim. Sci. Biotechnol..

[101] Biasato I., Renna M., Gai F., Dabbou S., Meneguz M., Perona G., Martinez S., Lajusticia A.C.B., Bergagna S., Sardi L. (2019). Partially defatted black soldier fly larva meal inclusion in piglet diets: Effects on the growth performance, nutrient digestibility, blood profile, gut morphology and histological features. J. Anim. Sci. Biotechnol..

[102] Chia S., Tanga C., Osuga I., Alaru A., Mwangi D., Githinji M., Dubois T., Ekesi S., Van Loon J., Dicke M. (2021). Black soldier fly larval meal in feed enhances growth performance, carcass yield and meat quality of finishing pigs. J. Insects Food Feed.

[103] Yu M., Li Z., Chen W., Rong T., Wang G., Li J., Ma X. (2019). Use of *Hermetia illucens* larvae as a dietary protein source: Effects on growth performance, carcass traits, and meat quality in finishing pigs. Meat Sci..

[104] Ipema A.F., Gerrits W.J., Bokkers E.A., Kemp B., Bolhuis J.E. (2021). Live black soldier fly larvae (*Hermetia illucens*) provisioning is a promising environmental enrichment for pigs as indicated by feed-and enrichment-preference tests. Appl. Anim. Behav. Sci..

[105] Cevolani D. (2004). Prontuario degli Alimenti per il Suino. 75 Schede per Valutare le Materie Prime.

[106] Schone F., Jahreis G., Lange R., Seffner W., Groppel B., Hennig A. (1990). Effect of varying glucosinolate and iodine intake via rapeseed meal diets on serum thyroid hormone level and total iodine in the thyroid in growing pigs. Endocrinol. Exp..

[107] Mejicanos G., Sanjayan N., Kim I.H., Nyachoti C.M. (2016). Recent advances in canola meal utilization in swine nutrition. J. Anim. Sci. Technol..

[108] Jansman A.J., Verstegen M.W.A., Huisman J., van den Berg J.W.O. (1995). Effects of hulls of faba beans (*Vicia faba* L.) with a low or high content of condensed tannins on the apparent ileal and fecal digestibility of nutrients and the excretion of endogenous protein in ileal digesta and feces of pigs. J. Anim. Sci..

[109] Svetina A., Jerković I., Vrabac L., Ćurić S. (2003). Thyroid function, metabolic indices and growth performance in pigs fed 00-rapeseed meal. Acta Vet. Hung..

[110] Lestingi A., Colonna M.A., Marsico G., Tarricone S., Facciolongo A.M. (2019). Effects of legume seeds and processing treatment on growth, carcass traits and blood constituents of fattening lambs. S. Afr. J. Anim. Sci..

[111] Batterham E.S., Saini H.S., Andersen L.M., Baigent R.D. (1993). Tolerance of growing pigs to trypsin and chymotrypsin inhibitors in chickpeas (*Cicer arietinum*) and pigeonpeas (*Cajanus cajan*). J. Sci. Food Agric..

[112] Jansman A.J.M., Huisman J., van der Poel A.F.B. (1993). Ileal and faecal digestibility in piglets of field beans (*Vicia faba* L.) varying in tannin content. Anim. Feed Sci. Technol..

[113] Flis M., Sobotka W., Purwin C., Zdunczyk Z. (1999). Nutritional value of diets containing field bean (*Vicia faba* L.) seeds with high or low proanthocyanidin levels for pig. J. Anim. Feed Sci..

[114] Dunshea F.R., Gannon N.J., van Barneveld R.J., Mullan B.P., Campbell R.G., King R.H. (2001). Dietary lupins (*Lupinus angustifolius* and *Lupinus albus*) can increase digesta retention in the gastrointestinal tract of pigs. Aust. J. Agric. Res..

[115] Stein H.H., Bohlke R.A. (2007). The effects of thermal treatment of field peas (*Pisum sativum* L.) on nutrient and energy digestibility by growing pigs. J. Anim. Sci..

[116] Owusu-Asiedu A., Baidoo S.K., Nyachoti C.M. (2002). Effect of heat processing on nutrient digestibility in pea and supplementing amylase and xylanase to raw, extruded or micronized pea-based diets on performance of early weaned pigs. Can. J. Anim. Sci..

[117] O’Doherty J.V., Keady U. (2001). The effect of expander processing and extrusion on the nutritive value of peas for pigs. Anim. Sci..

[118] Gdala J., Jansman A.J.M., Buraczewska L., Huisman J., van Leeuwen P. (1997). The influence of α-galactosidase supplementation on the ileal digestibilityof lupin seed carbohydrates and dietary protein in young pigs. Anim. Feed Sci. Technol..

[119] Christodoulou V., Ambrosiadis J., Sossidou E., Bampidis V., Arkoudilos J., Hucko B., Iliadis C. (2006). Effect of replacing soybean meal by extruded chickpeas in the diets of growing–finishing pigs on meat quality. Meat Sci..

[120] Partanen K., Alaviuhkola T., Siljander-Rasi H., Suomi K. (2003). Faba beans in diets for growing-finishing pigs. Agric. Food Sci..

[121] Skoufos I., Tzora A., Giannenas I., Bonos E., Papagiannis N., Tsinas A., Christaki E., Florou-Paneri P. (2016). Dietary inclusion of rapeseed meal as soybean meal substitute on growth performance, gut microbiota, oxidative stability and fatty acid profile in growing-fattening pigs. Asian J. Anim. Vet. Adv..

[122] Hansen J.Ø., Øverland M., Skrede A., Anderson D.M., Collins S.A. (2020). A meta-analysis of the effects of dietary canola/double low rapeseed meal on growth performance of weanling and growing-finishing pigs. Anim. Feed Sci. Technol..

